# Circulating microRNAs -192 and -194 are associated with the presence and incidence of diabetes mellitus

**DOI:** 10.1038/s41598-018-32274-9

**Published:** 2018-09-24

**Authors:** Andrea Jaeger, Lukas Zollinger, Christoph H. Saely, Axel Muendlein, Ioannis Evangelakos, Dimitris Nasias, Nikoleta Charizopoulou, Jonathan D. Schofield, Alaa Othman, Handrean Soran, Dimitris Kardassis, Heinz Drexel, Arnold von Eckardstein

**Affiliations:** 1 Institute for Clinical Chemistry, University Hospital Zurich, Zurich, Switzerland; 2 Centre for Integrative Human Physiology, University of Zurich, Zurich, Switzerland; 3 Vorarlberg Institute for Vascular Investigation and Treatment (VIVIT), Feldkirch, Austria; 4 Department of Medicine and Cardiology, Academic Teaching Hospital Feldkirch, Feldkirch, Austria; 5 Private University of the Principality of Liechtenstein, Triesen, Liechtenstein; 6 University of Crete Medical School and Institute of Molecular Biology and Biotechnology-FORTH, Heraklion, Greece; 7 Cardiovascular Trials Unit, The Old St Mary’s Hospital, Central Manchester University Hospitals, Manchester, United Kingdom; 8 Division of Cardiovascular Sciences, Cardiovascular Research Group, School of Medical Sciences, University of Manchester, Manchester, United Kingdom; 9 Drexel University College of Medicine, Philadelphia, PA USA

## Abstract

We sought to identify circulating microRNAs as biomarkers of prevalent or incident diabetes. In a pilot study of 18 sex- and age-matched patients with metabolic syndrome, nine of whom developed diabetes during 6 years of follow-up, an array of 372 microRNAs discovered significantly elevated serum levels of microRNAs -122, -192, -194, and -215 in patients who developed diabetes mellitus type 2 (T2DM). In two cross-sectional validation studies, one encompassing sex- and age-matched groups of patients with T2DM, impaired fasting glucose (IFG) and euglycemic controls (n = 43 each) and the other 53 patients with type 1 diabetes and 54 age- and BMI-matched euglycemic controls, serum levels of miR-192, miR-194, and mi215 were significantly higher in diabetic subjects than in probands with euglycemia or IFG. In a longitudinal study of 213 initially diabetes-free patients of whom 35 developed diabetes during 6 years of follow-up, elevated serum levels of microRNAs 192 and 194 were associated with incident T2DM, independently of fasting glucose, HbA1c and other risk factors. Serum levels of miR-192 and miR-194 were also elevated in diabetic Akt2 knockout mice compared to wild type mice. In conclusion, circulating microRNAs -192 and -194 are potential biomarkers for risk of diabetes.

## Introduction

Diabetes is characterized by a relative or absolute lack of insulin that results in hyperglycaemia. Diabetes mellitus type 1 (T1DM) is caused by the autoimmune destruction of the pancreatic beta cells whereas Diabetes mellitus type 2 (T2DM) is characterized by insulin resistance that is not sufficiently compensated by insulin secretion of the pancreatic beta-cells. At diagnosis of T2DM, patients already display metabolic alterations and many of them also have developed complications including renal disease, retinopathy, polyneuropathy, and coronary artery disease (CAD)^1–3^. The delay or even prevention of these complications require early identification of patients at risk for diabetes, ideally before or independently of hyperglycemia. The combination of lifestyle factors, anthropometric (BMI, waist to hip ratio, blood pressure) and metabolic biomarkers (glucose, glycated hemoglobin (HbA1c), triacylglycerol, high density lipoprotein (HDL)) identify individuals at risk of diabetes^4^. However, the prognostic value of these risk factors is limited^5^. As the consequence, novel biomarkers identifying patients at risk for diabetes are searched by both candidate-driven and screening approaches.

Micro-ribonucleic acids (miRNAs) circulating in blood are accessible for biomarker discovery by screening approaches^6,7^. MiRNAs are small non-coding RNAs that modify gene expression at the post-transcriptional level. They bind to a complementary site in the 3′ untranslated region of their target mRNAs and inhibit their translation or direct their degradation^8,9^. Thereby, miRNAs regulate many biological processes such as cellular differentiation, proliferation, apoptosis, and metabolic homeostasis^10^. Some miRNAs are released into the circulation either passively upon cell injury and death or actively for intercellular communication^11^. In the bloodstream, miRNAs are protected from degradation by RNAses because they are packaged into exosomes, microparticles, apoptotic bodies, or lipoproteins, or linked to RNA-binding proteins^12^. Interestingly, the serum concentrations of specific miRNAs differ with pathological conditions so that miRNAs are increasingly explored as potential biomarkers for many diseases^13^.

Previous miRNA expression profiling studies aiming at the identification of novel biomarkers for T2DM, were performed in whole blood^14,15^, plasma^16–18^, serum^19,20^, or exosomes^21^ Zhu *et al*.^13^ and Villard *et al*.^22^ performed meta-analyses to identify consistently dysregulated miRNAs. Among 18 miRNA profiling studies Zhu *et al*.^13^ identified seven circulating miRNAs (miR-29a, miR-34a, miR-375, miR-103, miR-107, miR-132, miR-142-3p and miR-144) as potential biomarkers of T2DM. In their meta-analysis, Villard *et al*.^22^ included eight studies that compared pre-diabetic patients vs control subjects and 14 studies that compared diabetic vs BMI-matched non-diabetic subjects. They found ten miRNAs altered in blood of patients suffering from T2DM (miR-320a, miR-222, miR-29a, miR-27a, miR-375, miR-197, miR-20b, miR-17, miR-652, and miR-142-3p). Only three miRNAs (miR-142-3p, miR-29a and miR-375) were hence the common finding of the two meta-analyses. Of note, all previous profiling studies, except the prospective Bruneck Study^18,23^, had cross-sectional or case-control designs and hence were unable to investigate the prognostic value of circulating miRNAs towards the development of new-onset T2DM. The current study addresses this gap and aims at identifying circulating miRNAs that are associated with present or incident diabetes.

## Results

### Discovery of circulating miRNAs associated with the development of future type 2 diabetes

To discover circulating miRNAs associated with the risk of diabetes, we compared the expression of 372 miRNAs between sex and age-matched patient groups from the VIVIT cohort: All patients had metabolic syndrome at baseline while nine patients developed diabetes during a 6-year observation period (incident diabetes) and nine did not (no incident diabetes). Circulating miRNA profiles were recorded in four sera per patient that were collected at baseline as well as at the 2-, 4-, and 6-year visits. At baseline, the patient groups did not differ significantly with respect to sex, age, BMI, fasting glucose, HbA1c, or presence of metabolic syndrome or cardiovascular disease (Supplemental Table S1). MiRNAs were selected as candidate biomarkers for incident diabetes if they were significantly up- or downregulated in sera obtained from at least one visit, *and* showed a consistent up- or downregulation in all follow-up samples. According to these criteria, the serum level of miR-10b was lower in patients with incident diabetes compared to subjects without incident diabetes, whereas the concentrations of miR-22*, miR-122, miR-192, miR-193b, miR-194, and miR-215 were higher (Supplemental Fig. S1 and Supplemental Table S2). In principle the same result was obtained by an alternative analysis in which we compared the individual time points of patients with incident diabetes with the aggregated value from all non-diabetic time points (Supplemental Fig. S2). These associations were reproduced by individual quantitative real-time PCR (qRT-PCR) assays for miR-122, miR-192, miR-194 and miR-215 but not for miR-10b and miR-22*. The latter two miRNAs as well as miR-193b, whose concentrations were close to the limit of detection, were excluded from the subsequent validation studies.

### Circulating miR-192, miR-194, and miR-215 are elevated in sera of patients with the presence of both type 2 diabetes and type 1 diabetes

**Figure 1:**
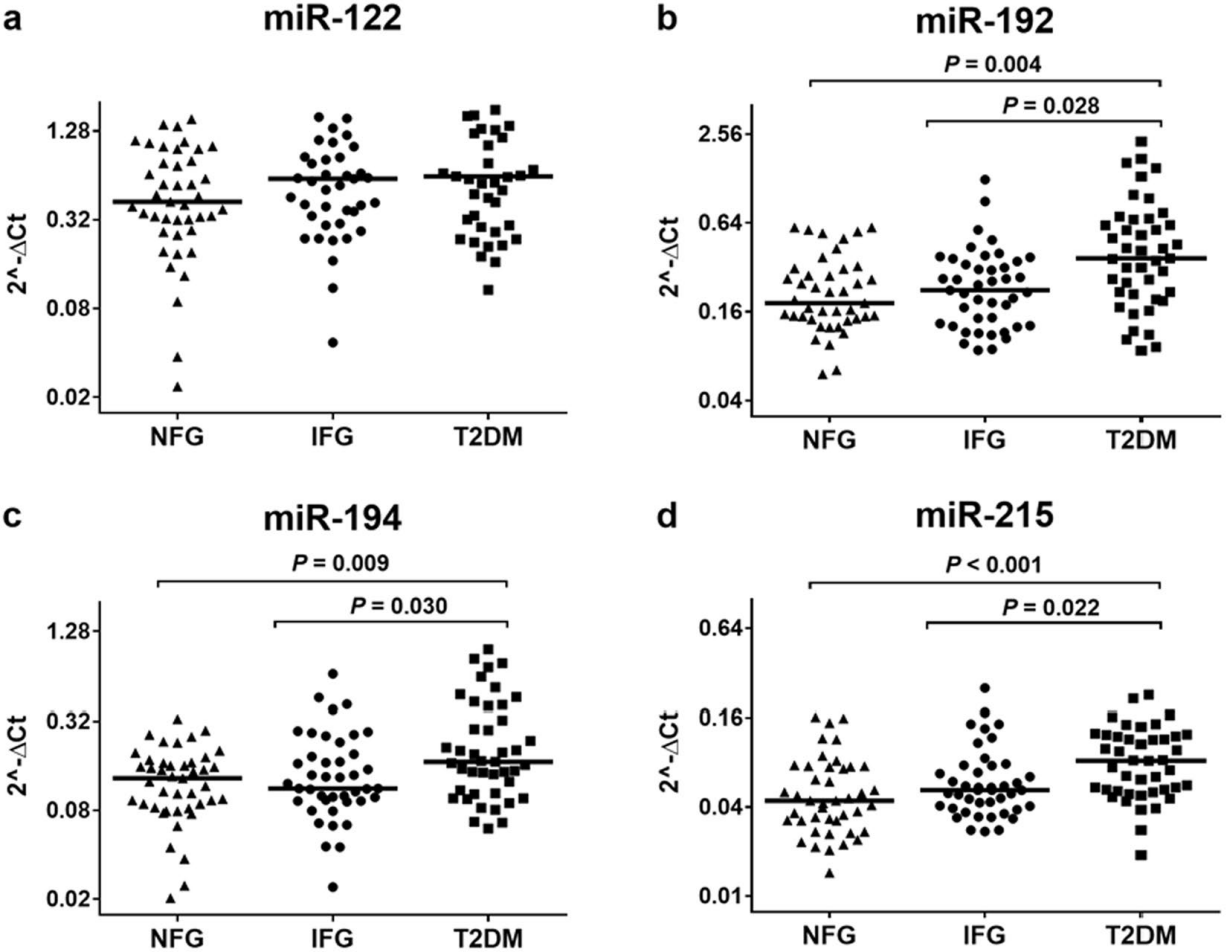
Serum levels of miR-122 (**a**), miR-192 (**b**), miR-194 (**c**), and miR-215 (**d**) in subjects with NFG, IFG, and manifest type 2 diabetes. The miRNAs -192, -194 and -215 were significantly higher in patients with manifest diabetes as compared to patients with NFG or IFG. Circulating miR-122 did not significantly differ between any groups. To compare miRNA levels between patient groups the Kruskal-Wallis test was run followed by Dunn’s procedure for pairwise comparisons and a Bonferroni correction for multiple testing. T2DM: type 2 diabetes mellitus. Horizontal lines show the medians.

In a first cross-sectional validation, serum levels of miR-122, miR-192, miR-194, and miR-215 were investigated by individual qRT-PCR assays for their association with glycemic stages in patients of the VIVIT cohort. Because of the selection criteria, the sex- and age-matched 43 euclycemic control subjects, 43 patients with impaired fasting glucose (IFG), and 43 patients with manifest T2DM significantly differed by the prevalence of metabolic syndrome as well as frequency distributions of BMI, fasting glucose, HbA1c, C-peptide and insulin (Supplemental Table S3). Kruskal-Wallis H test revealed significantly higher levels of miR-192, miR-194 and miR-215 in sera of patients with manifest T2DM as compared to patients with euglycemic controls or IFG (manifest T2DM vs NFG: P = 0.004 for miR-192, P = 0.009 for miR-194, P < 0.001 for miR-215; manifest T2DM vs IFG: P = 0.028 for miR-192, P = 0.030 for miR-192, P = 0.022 for miR-215) (Fig. 1, Supplemental Table S4). The serum concentration of miR-122 did not significantly differ between any groups.

**Figure 2:**
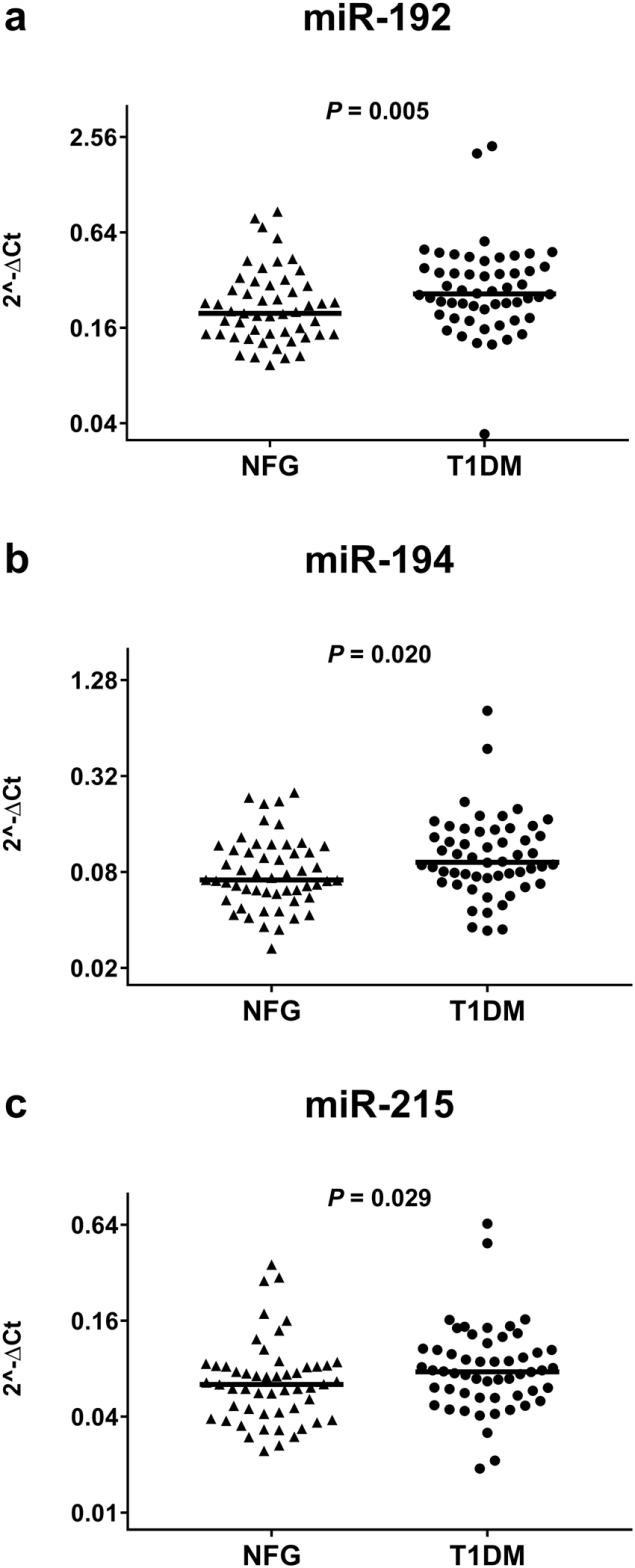
Serum levels of miR-192 (**b**), miR-194 (**c**), and miR-215 (**d**) in subjects with NFG or type 1 diabetes. Serum levels of miRNA -192 were significantly higher in patients with T1DM diabetes as compared to patients with NFG. To compare miRNA levels between patient groups the Kruskal-Wallis test was run followed by Dunn’s procedure for pairwise comparisons and a Bonferroni correction for multiple testing. T1DM: type 1 diabetes mellitus. Horizontal lines show the medians.

We next compared the expression of miR-192, miR-194, and miR-215 in sera of 54 patients with T1DM and 53 non-diabetic control subjects. The two groups resembled each other by age, gender, and BMI but differed significantly by higher eGFR, higher plasma levels of glucose and HDL cholesterol as well as lower plasma levels of total and LDL cholesterol as well as triglycerides in T1DM patients. Apart from insulin therapy, the T1DM patients were more frequently treated with statins and ACE-inhibitors (Supplemental Table S5). The Mann-Whitney U test revealed significantly higher serum levels of miR-192 (*P* = 0.005), miR-194 (*P* = 0.020), and miR-215 (*P* = 0.026) in sera of subjects with T1DM compared to subjects with NFG. (Fig. 2, Supplemental Table S6).

### Circulating miR-192 and miR-194 predict the development of future type 2 diabetes

**Figure 3:**
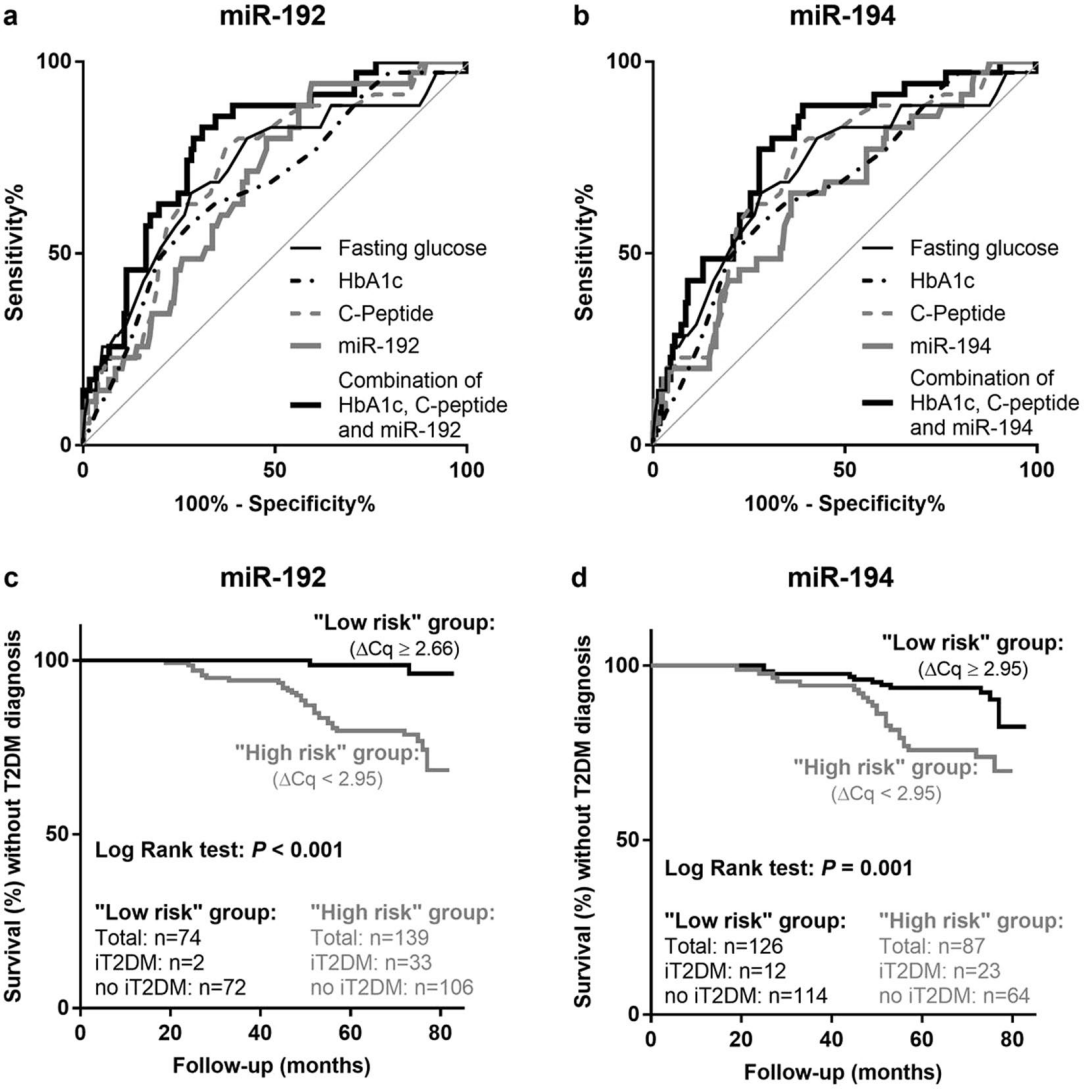
Associations of circulating miR-192 (**a**,**c**) and miR-194 (**b**,**d**) with incident diabetes as analyzed by C-statistics (**a**,**b**) or Kaplan-Meier survival functions (**c**,**d**). The ROC curves of the four most accurate predictors are presented (**a** and **b**). The combination of miR-192 with HbA1c and C-peptide resulted in the highest AUC and hence was the best model to predict incident diabetes (**a**). The sensitivity, specificity, and AUC values for the predictors are summarized in Table 3. Kaplan-Meier survival curves show the time to diabetes-diagnosis for patients with low versus high serum levels of miR-192 (**c**) and miR-194 (**d**). Low risk and high risk refer to optimal cutoff points calculated with the Youden-Index (2.66 ΔCq for miR-192, 2.95 ΔCq for miR-194). iT2DM: incident type 2 diabetes mellitus.

The association of candidate miRNAs with incident T2DM was assessed in 213 patients of the VIVIT cohort without manifest T2DM at baseline. The miRNA levels were compared between patients who developed T2DM during the 6-year observation period (n = 35) and those who did not (n = 178). Patients with incident T2DM differed significantly from patients without incident T2DM by higher concentrations of fasting glucose, HbA1c, C-peptide, triglycerides (Supplemental Table S7) as well as higher levels of miR-192 and miR-194 but not by miR-122 and miR-215 levels (Fig. 3, Supplemental Table S8).

Univariate logistic regression revealed that miR-192 and miR-194, along with fasting glucose, HbA1c and C-peptide were significant predictors for the development of T2DM (Table 1). This finding persisted in multivariate logistic regression models, when either the significant variables from the univariate models (fasting glucose, HbA1c, C-peptide; Table 2) or classical risk factors (sex, age, metabolic syndrome; Supplemental Table S9) were set as covariates. The triple combination of HbA1c (OR 3.08, 95%CI 1.27–7.50; P = 0.013), C-peptide (OR 1.94, 95%CI 1.18–3.19; P = 0.009) and miR-192 (OR 1.84, 95%CI 1.23–2.75; P = 0.003) performed best and explained 23.8% (Nagelkerke R^2^) of the variance (Model 2a in Table 2). Interestingly, adjustment for miR-194 made miR-192 a non-significant predictor and vice versa, while each regression model kept its statistical significance. This was probably due to the strong correlation between these two co-expressed miRNAs (see below)^24^. MiR-122 and miR-215 were not associated with incident T2DM.

**Table 1:** Risk of incident diabetes according to different univariate logistic regression models.

Standardized covariates	OR (per increase of 1 SD of the biomarker)	(95% CI)	*P*-value	*Nagelkerke R*^2^ (%)
Sex	0.66	(0.28–1.53)	0.329	0.8
Age	1.01	(0.70–1.44)	0.979	<0.1
MetS (ATPII)	1.97	(0.91–4.30)	0.087	2.1
**Fasting glucose**	**4**.**26**	**(1**.**74–10**.**42)**	**0**.**002**	**8**.**2**
**HbA1c**	**3**.**83**	**(1**.**65–8**.**90)**	**0**.**002**	**8**.**0**
**C-peptide**	**2**.**16**	**(1**.**41–3**.**32)**	**<0**.**001**	**10**.**7**
coronary stenosis > 50%	1.702	(0.79–3.69)	0.177	1.5
History of myocardial infarction	1.336	(0.62–2.89)	0.462	0.4
History of stroke	4.078	(0.87–19.09)	0.074	2.2
miR-122	1.07	(0.74–1.53)	0.732	0.1
**miR-192**	**1**.**95**	**(1**.**34–2**.**83)**	**<0**.**001**	**11**.**0**
**miR-194**	**1**.**91**	**(1**.**29–2**.**83)**	**0**.**001**	**9**.**7**
miR-215	0.89	(0.60–1.31)	0.557	0.03

**Table 2:** Risk of incident diabetes according to different multivariate logistic regression models.

	Standardized covariates	OR (per increase of 1 SD of the biomarker)	(95% CI)	*P*-value	*Nagelkerke R*^2^ (%)
Model 1a	Fasting glucose	2.42	(0.91–6.39)	0.076	21.3
	C-peptide	1.92	(1.18–3.12)	0.008	
	miR-192	1.65	(1.11–2.45)	0.014	
Model 1b	Fasting glucose	2.88	(1.09–7.63)	0.033	20.4
	C-peptide	1.84	(1.13–3.00)	0.015	
	miR-194	1.58	(1.05–2.39)	0.029	
Model 2a	HbA1c	3.08	(1.27–7.50)	0.013	23.8
	C-peptide	1.94	(1.18–3.19)	0.009	
	miR-192	1.84	(1.23–2.75)	0.003	
Model 2b	HbA1c	2.90	(1.21–6.99)	0.017	21.4
	C-peptide	1.93	(1.18–3.16)	0.009	
	miR-194	1.65	(1.09–2.52)	0.019	

**Figure 4:**
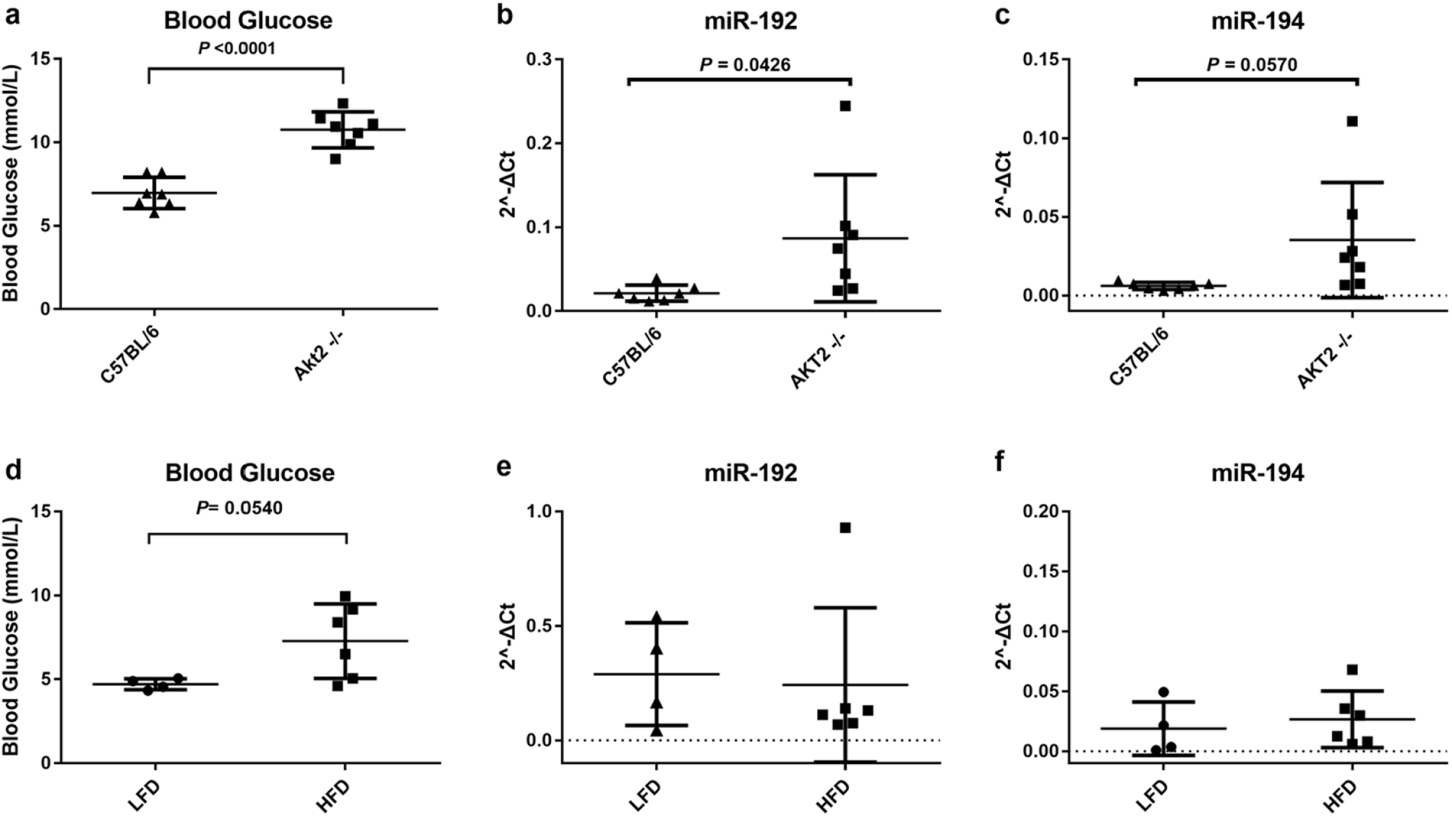
Serum levels of glucose, miR-192 and miR-194 in mouse models of diabetes. Glucose concentrations were higher in Akt knockout mice compared to wild type mice (**a**) and in apoE3Leiden*CETP transgenic mice fed with a high fat diet versus low fat diet (**d**). Circulating miR-192 (**b**,**c**) and miR-194 (**e**,**f**) were elevated in Akt2 knockout mice compared to wild type mice but not in apoE3Leiden*CETP transgenic mice fed with a high fat diet versus low fat diet. To compare miRNA levels between groups of mice the Student’s t-test was performed. C57BL/6: wild type mice; Akt2−/−: Akt2 knockout mice; apoE3L*CETP: apoE3Leiden*CETP transgenic mice; LFD: low fat diet; HFD: high fat diet.

The ability of the candidate miRNAs to predict incident T2DM was also evaluated by means of C-statistics and compared to the performance of the other significant predictors (glucose, HbA1c, C-peptide) (Fig. 3, Table 3). The highest area under the curve (AUC) and hence best predictive values were observed for fasting glucose (0.72, 95% CI: 0.62–0.82) and C-peptide (0.72, 95% CI: 0.63–0.81). HbA1c (0.67, 95% CI 0.58–0.77), miR-192 (0.68; 95% CI: 0.59–0.77) and miR-194 (0.65, 95% CI: 0.56–0.75) showed slightly lower but similar AUCs. In line with the results of the logistic regression, the combination of miR-192, HbA1c and C-peptide performed best in predicting T2DM. This triple combination increased the AUC compared to fasting glucose or C-peptide alone by 0.07 to 0.79 [95% CI: 0.73–0.84] (Fig. 4, Table 3). This increase was statistically significant when compared to the AUC of C-peptide (*P* = 0.012) but not when compared to the AUC of fasting glucose (*P* = 0.172). MiR-192 contributed 0.05 to this increase. Then, optimum cutoffs to maximize sensitivity and specificity were calculated with the Youden Index^25^ and were 2.66 ΔCq for miR-192 and 2.95 ΔCq for miR-194. For fasting glucose and HbA1c the upper limit of the normal range according to the American Diabetes Association (ADA) was used as the cutoff (glucose: 5.60 mmol/l, HbA1c: 5.70% [~39 mmol/mol]), because the cutoffs calculated with the Youden index (glucose: 5.52 mmol/l, HbA1c: 5.85% [~40 mmol/mol]) were very close. Based on the optimal cutoff, the triple combination of miR-192, HbA1c and C-peptide predicted the development of T2DM with a sensitivity of 83% and a specificity of 69%. Moreover, integrated discrimination improvement (IDI), that is a measure of improved risk prediction of a new model compared to a reference model^26^, was significantly different from zero when the triple combination was compared to glucose or HbA1c alone (*P* = 0.015 and *P* < 0.001). Relative IDI was 106% when glucose was used as the reference model and 190% when HbA1c was used. No other combination further improved the predictive performance.

**Table 3:** Prognostic performance of circulating miRNAs and established biomarkers towards incident diabetes.

	C-statistics			Sensitivity and Specificity			IDI (integrated discrimination improvement)			
	AUC	(95% CI)	*P-*value	Sensitivity	Specificity	Specificity at 80% sensitivity	absolute IDI	(95% CI)	*P*-value	relative IDI
**Individual parameters**										
Fasting glucose	0.72	(0.62–0.82)	<0.001	60%	74%	57%				
HbA1c	0.67	(0.58–0.77)	0.001	69%	51%	39%				
C-peptide	0.72	(0.63–0.81)	<0.001	77%	63%	60%				
miR-192	0.68	(0.59–0.77)	0.001	94%	40%	48%				
miR-194	0.65	(0.56–0.75)	0.004	66%	64%	40%				
**Combined parameters**										
Fasting glucose	0.72	(0.62–0.82)	<0.001	60%	74%	57%	reference			
Glucose, C-peptide	0.76	(0.70–0.81)	<0.001	89%	55%	57%	0.039	(0.000–0.039)	0.050	50%
Glucose, miR-192	0.74	(0.64–0.84)	<0.001	66%	82%	51%	0.047	(0.006–0.053)	0.026	60%
Glucose, miR-194	0.76	(0.66–0.85)	<0.001	54%	91%	55%	0.045	(0.007–0.051)	0.021	58%
Glucose, C-peptide, miR-192	0.77	(0.71–0.83)	<0.001	77%	70%	61%	0.080	(0.024–0.104)	0.005	103%
Glucose, C-peptide, miR-194	0.78	(0.72–0.83)	<0.001	63%	84%	64%	0.070	(0.022–0.092)	0.005	91%
HbA1c	0.67	(0.58–0.77)	0.001	69%	51%	39%	reference			
HbA1c, C-peptide	0.75	(0.69–0.81)	<0.001	77%	67%	63%	0.050	(0.011–0.061)	0.011	90%
HbA1c, miR-192	0.75	(0.67–0.84)	<0.001	74%	70%	48%	0.072	(0.022–0.095)	0.005	131%
HbA1c, miR-194	0.73	(0.65–0.82)	<0.001	86%	51%	53%	0.059	(0.014–0.073)	0.010	107%
HbA1c, C-peptide, miR-192	0.79	(0.73–0.84)	<0.001	83%	69%	71%	0.105	(0.043–0.148)	<0.001	190%
HbA1c, C-peptide, miR-194	0.78	(0.72–0.83)	<0.001	89%	61%	69%	0.087	(0.035–0.122)	0.001	158%

AUC with 95% CI, sensitivity, specificity, and IDI (integrated discrimination improvement) are presented in table. IDI is a measure of improved risk prediction of a new model compared to a reference model. Sensitivity and specificity were calculated based on optimum cutoffs calculated with the Youden Index^25^.

To further assess the predictive potential of the candidate miRNAs, patients were assigned to either a «high risk» or a «low risk» group for future T2DM depending on whether their miRNA levels were below or above the optimum cutoff calculated using the Youden Index^25^. Then, the time to T2DM diagnosis was compared between «high risk» and «low risk» patients by means of Kaplan-Meier estimation and log-rank test. «High risk» patients with elevated levels of miR-122, miR-192 and/or miR-194 showed significantly different disease-free survival distributions compared to «low risk» patients with low circulating miRNAs (χ^2^(1) = 3.96, *P* < 0.047 for miR-122; χ^2^(1) = 14.55, *P* < 0.001 for miR-192; χ^2^(1) = 10.84, P = 0.001 for miR-194) (Fig. 3, Table 3). However, no significant change of survival distributions was found for low versus high miR-215 concentrations (χ^2^(1) = 0.446, *P* = 0.504; Supplemental Fig. S3).

### Correlation of circulating miRNAs with anthropometric and metabolic measures

A Spearman’s rank-order regression analysis (Table 4) revealed medium to strong positive correlations between serum levels of miR-122, miR-192, miR-194, and miR-215 in both the VIVIT and Manchester cohorts. In the VIVIT cohort all four miRNAs correlated with the activity of the liver enzymes alanine transaminase (ALT), aspartate transaminase (AST) and gamma glutamyl transferase (GGT). In addition, miR-192 and miR-194 correlated with C-peptide, insulin, and homeostasis model assessment-index ratio (HOMA-IR). In the VIVIT cohort but not in the Manchester cohort, miR-192 and miR-215 correlated with estimated glomerular filtration rate (eGFR) and inversely with cystatin C levels.

**Table 4:** Spearman correlation between circulating miRNAs, anthropometric and laboratory parameters (at study baseline).

	patient number	miR-122		miR-192		miR-194		miR-215	
Age	213	**−0**.**301**	***	**−0**.**248**	***	**−0**.**231**	**	**−0**.**262**	***
BMI	213	0.125		0.128		0.095		0.087	
**Glucose homeostasis**									
Fasting glucose	213	0.075		**0**.**142**	*	0.081		**−**0.028	
HbA1	213	**−**0.054		0.034		0.017		**−**0.060	
C-Peptide	213	**0**.**232**	**	**0**.**237**	***	**0**.**280**	***	0.026	
Insulin	194	0.121		**0**.**164**	*	**0**.**165**	*	0.038	
HOMA-beta	194	0.112		0.074		**0**.**146**	*	0.042	
HOMA-IR	194	0.129		**0**.**183**	*	**0**.**170**	*	0.042	
**Kidney function**									
Creatinine	207	**−**0.034		**−**0.103		0.012		**−0**.**176**	*
Cystatine C	207	**−**0.080		**−0**.**232**	**	**−**0.071		**−0**.**205**	**
eGFR (CKD-EPI)	207	**0**.**189**	**	**0**.**217**	**	0.132		**0**.**292**	***
**Liver values**									
AST	191	**0**.**325**	***	**0**.**231**	**	**0**.**267**	***	**0**.**229**	**
ALT	191	**0**.**357**	***	**0**.**297**	***	0.154	*	**0**.**396**	***
GGT	191	**0**.**390**	***	**0**.**307**	***	**0**.**271**	***	**0**.**218**	**
**Lipid values**									
Triacylglycerol	123	**0**.**356**	***	**0**.**215**	*	**0**.**273**	**	0.080	
Cholesterol	123	0.026		**−**0.031		0.055		0.067	
HDL	123	**−0**.**187**	*	**−**0.069		**−**0.094		0.045	
LDL	123	**−**0.011		**−**0.169		**−**0.110		**−**0.010	
**miRNAs**									
miR-122	213			**0**.**565**	***	**0**.**591**	***	**0**.**434**	***
miR-192	213					**0**.**705**	***	**0**.**450**	***
miR-194	213							**0**.**317**	***

CKD-EPI: Chronic-Kidney-Disease-Epidemiology, *P < 0.05; **P < 0.01; ***P < 0.001.

### MiRNAs-192 and -194 in sera of mice with diabetes or metabolic syndrome

Finally, we investigated whether serum levels of miR-192 or miR-194 are also altered in two mouse models of T2DM and insulin resistance of different pathogenic origin (Fig. 4)^27^. Compared to wild type mice, protein kinase B (Akt2)−/− mice^27^ showed 25% and 400–1000% higher plasma concentrations of glucose (Fig. 4A) and insulin (Supplementary Fig. 4A), respectively, as well as significantly elevated serum levels of both miR-192 or miR-194 (Fig. 4B,C). Upon a high fat diet (HFD) but not upon a low fat diet (LFD), mice expressing the mutant apoE3Leiden and CETP (E3L*CETP) develop obesity, fatty liver, and insulin resistance^27^. Glucose and insulin levels were elevated in half of the mice (p = 0.054, Fig. 4D, and p = 0.0121, Supplementary Fig. 4B, respectively) However, serum levels of miR-192 and miR-194 did not differ between HFD and LFD mice (Fig. 4E,F).

## Discussion

By combining a biomarker discovery study with three targeted validation studies, we found elevated serum levels of miR-192, miR-194 and miR-215 associated with the presence of both T1DM and T2DM, and miR-192 and miR-194 associated with the incidence of T2DM. The association of miR-192 and miR-194 with incident T2DM showed similar strength as fasting glucose, HbA1c, and C-peptide. With AUCs ranging between 0.67 and 0.72, none of the five individual predictors was good enough to serve as a clinically useful prognostic biomarker *per se*, but the combination of miR-192, HbA1c, and C-peptide nearly reached the threshold of clinical utility^26^ and predicted the disease with an AUC of 0.79 (95%CI [0.73–0.84]) in a period of at least 2–6 years before onset. Of note, serum levels of miR-192 and miR-194 were also elevated in Akt2−/− mice^28^ which have a diabetes-like syndrome due to blocked insulin signaling but not in E3L*CETP mice which develop a diabetes-like syndrome upon fat-overload^27^.

The finding of both miR-192 and miR-194 testifies the quality of our unbiased approach because they are expressed by the same precursor miRNA (miR-192/–194 pri-miR)^24^. Five other but smaller and non-prospective miRNA profiling studies found associations of miR-192 or miR-194 serum levels with glycemic stage^15,16,29–31^: One study found miR-192 increased in sera of patients with prediabetes but not altered in sera of patients with manifest T2DM. Interestingly, the authors also reported that miR-192 decreases to normal levels in both insulin resistant humans and mice upon sustained physical exercise normalizing the metabolic phenotype^29^. A similar observation was made upon treatment of pre-diabetic patients with 2000 U vitamin D/day: Circulating levels of miR-192 dropped upon treatment with vitamin D but not placebo and the change in miR-192 levels correlated with the changes of glucose levels^32^. Circulating miR-194 seems also to be associated with the changes of glucose levels: A study following metabolic syndrome patients over 1 year showed that miR-194 was differentially expressed in patients with variable fasting glucose levels compared to patients with stable glucose levels^33^. An observational study reported increased levels of miR-194 in polycystic ovary syndrome patients with impaired glucose metabolism compared to patients with normal glucose metabolism^30^. Likewise miR-192 levels were found increased in full blood samples of patients or rats with T2DM as compared to normoglycemic controls^15^. By contrast to those and our studies, Ortega *et al*.^16^ and Yang *et al*.^31^ reported decreased rather than elevated miR-192 serum levels in patients with manifest T2DM. Ortega *et al*.^16^ also found, that miR-192 concentration increased upon treatment with metformin. In agreement with our data, a higher abundance of miR-192, miR-194 and miR-122 was associated with greater insulin resistance in 2317 non-diabetic subjects of the Framingham Heart Study^34^. Moreover, a meta-analysis of several profiling studies identified miR-192 upregulated in liver of diabetic subjects compared to liver of nondiabetic subjects^13^.

The observational design of our study allows any conclusion on neither the origin of miR-192 and miR-194 in the circulation nor on the pathogenic relationship between their serum levels with prevalent or incident diabetes. Due to the systemic nature of diabetes and its complications, miRNAs may be released from several organs in response to cellular stress, injury, or death. MiR-192 and miR-194 are expressed by many organs that either play a role in the pathogenesis of T2DM (e.g. beta cells or liver)^30,35,36^ or are affected by diabetic complications, sometimes even before the onset of manifest T2DM (e.g. liver or kidney)^30,35,36^. Thus, the association of miR-192 and miR-194 may reflect causality, reverse causality, or an indirect bystander relationship. Supporting cause- or effect-relationships with insulin action and glucose utilization, we and others^30,34–36^ found significant correlations of miR-192 and miR-194 levels with the HOMA-IR index. Also, in support of direct relationships miR-192 was found among the top ten most abundant miRNAs in pancreatic beta-cells^37^ indicating a potential relevance of this miRNA for beta cell function and survival. This may be reflected by the association of miR-192 and miR194 with T1DM. In line with this, miR-192 targets the mRNA of the transcription factor Rfx6, which regulates islet formation and insulin production^38^. Serum levels of miR-194, were associated with residual beta cell function in children with T1DM^39^. MiR-192 and miR-194 are also expressed by liver, skeletal muscle and adipocytes where they were found to regulate insulin signaling^15,30,40,41^. MiR-194 regulates the phosphorylation of AKT, glycogen synthase kinase 3 (GSK3) as well as oxidative phosphorylation in skeletal muscle cells^40^. In rats, miR-194 was found to promote hyperglycemia by suppressing IGF-1 receptor expression and the phosphoinositide-3-kinase (PI3K)/AKT signal pathway in skeletal muscle^41^. This interaction of Akt with miR-192 and miR-194 may be reflected by the elevated serum levels of miR-192 and miR-194 in sera of Akt2−/− mice.

Three recent studies revealed elevated miR-192 in liver tissue and serum of patients with non-alcoholic fatty liver disease (NAFLD) and non-alcoholic steatohepatitis (NASH)^42–44^, which are both risk factors of incident T2DM and comorbidities of prevalent T2DM. In our cohort, serum levels of miR-192, miR-194, and miR-215 correlated with the serum activities of liver function parameters AST, ALT, and GGT as well as serum levels of triglycerides. However, feeding of E3L*CETP mice with HFD which induces an NAFLD-like phenotype with insulin-resistance, hyperglycemia, hyperlipidemia and hepatic steatosis^27^ did not lead to any changes in serum levels of miR-192 or miR-194. This contradiction may reflect that NAFLD can be both the cause and consequence of insulin resistance^45^. On the one hand hyperinsulinemia compensating for insulin resistance promotes lipogenesis in the liver by both Akt2-dependent and Akt2-independent signaling events. On the other hand, the intracellular increase of lipids promote pro-inflammatory signaling cascades such as NF-KB, JNK, and SOCS as well as activation of the inflammasome which inhibit the insulin-receptor signaling towards gluconeogenesis and glycogen synthesis^45^. The discrepant findings of elevated miR-192 and miR194 in akt2−/− mice but no alteration in HFD fed E3L*CETP mice may hence reflect the different pathogenesis of hyperglycemia in these mice: The lack of Akt2 causes insulin resistance however without promoting lipogenesis, steatosis and dyslipidemia, because some lipogenic effects of insulin involve signaling via Akt2^46^. In apoE3L*CETP mice steatosis is promoting insulin resistance^46^. In fact, functional studies point to the involvement of miR-192 or miR-194 in the pathogenesis of NASH. MiR-192 was found induced by docosahexaenoic acid in hepatocytes where it modulates lipid metabolism by targeting caveolins, fatty acid elongases, and the VLDL receptor^36^. Overexpressing or inhibiting miR-192 in the liver resulted in changes of total triacylglycerol accumulation^36^. MiR-194 inhibits the inflammatory toll-like receptor 4 pathway by targeting tumor necrosis factor receptor-associated factor 6 (TRAF6)^47^ and prevents stellate cell activation by targeting rac 1^48^. MiR-192 is known to be upregulated by TGF-β1^49^, a key fibrogenic cytokine in hepatic stellate cells.

As bystanders rather than cause or effect of diabetes, elevated miR-192 and miR-194 levels may be caused by co-morbidities of diabetes and prediabetes. Notably nephropathy^50,51^ has been repeatedly associated with altered miR-192 serum levels, which in the VIVIT cohort correlated with two biomarkers of kidney function, namely eGFR and cystatin C (Table 4). Of note we did not observe this association in the probands of the Manchester cohort who were younger and had normal kidney function. In the kidney, miR-192 is regulated by TGF-β1 upon high blood glucose levels. Moreover, miR-192 was found to play important roles in renal fibrosis, a key feature in diabetic nephropathy^52–57^. However, controversial data were reported on the association of miR-192 with nephropathy^34^. Chien *et al*.^50^ found higher miR-192 levels in patients with overt proteinuria group compared to those with microalbuminuria. In contrast, Ma *et al*.^51^ found lower levels of miR-192 in patients with macroalbuminuria compared to those with microalbuminuria or normoalbuminuria. These contradictory results may be due to differential expression of miR-192 that depend on the stage of renal injury and/or the cell type-specific response of miR-192 to TGF-β1^34^. In line with this hypothesis, miR-192 was increased in mouse mesangial cells treated with TGF-β1 and in renal glomeruli from mouse models of diabetes depicting early stages of diabetic nephropathy (streptozotocin-injected type-1 diabetic mice and type-2 diabetic *db/db* mice) compared to nondiabetic control mice^49,55,57^. Conversely, miR-192, is downregulated in models of more severe or late-stage diabetic nephropathy in diabetic ApoE-deficient mice^54^ and in biopsies from patients in late stages of diabetic nephropathy^58^. MiR-192 was also reported in some studies of cultured proximal tubular epithelial cells treated with high glucose or TGF-β1, which was associated with increased fibrosis^54,58^. Tubular damage and necrosis/apoptosis are usually observed in the later stages of diabetic nephropathy, which could lead to decreases in miRNA levels.

Our study has strengths and limitations. One strength is the combination of comprehensive miRNA profiling in a discovery study with validation by both cross-sectional and longitudinal cohort studies as well as animal models. Only three other studies reported on the associations of circulating miRNAs with incident T2DM^18,23,59,60^, while the vast majority of the studies only used cross-sectional validation. Of those investigating incident T2DM, the Bruneck study found lower levels of miR-15a, miR-29b, miR-126, and miR-223, as well as elevated levels of miR-28–3p and miR-122 to be associated with incident T2DM^18,23^. Flowers *et al*.^59^ found six miRNAs to be associated with incident T2DM in an Asian Indian population: miR-122, miR-15a, miR-197, miR-320a, miR-423, and miR-486. De Candia *et al*.^60^ investigated a small group of patients with IFG that progressed to T2DM (n = 4) or not (n = 5) and identified miR-143-3p, miR-320b and miR-320c to be differentially expressed. Thus, only miR-15a and miR-122 have been repeatedly been associated with incident T2DM. Importantly, we also identified miR-122 as a potential biomarker of incident T2DM in our discovery study. In our validation studies, elevated miR-122 levels were significantly associated with a shorter time to T2DM diagnosis when using Kaplan-Meier estimation (Supplemental Figure S3), but there was neither any association with glycemic states nor with incident T2DM when logistic regression analysis was applied. Of note, miR-192 was not included in the studies of Flowers *et al*.^59^ and Candia *et al*.^60^ while miR-194 was not included in any of the three studies investigating incident T2DM. Another strength of the present study is the biannual recall of probands for extensive examination including oral glucose tolerance testing (oGTT) that makes the rule-in and rule-out of incident T2DM more accurate than follow-up by phone call interviews or registries.

One limitation is the investigation of patients who had either suspected or established CAD and were therefore at higher risk to develop T2DM than individuals from the general population. However, neither in the discovery study (Supplemental Table S1) nor in the validation studies (Supplemental Tables S3 and S7), the patient groups differed significantly by prevalence of acute myocardial infarction (AMI) or stroke as well as the presence of significant coronary stenosis. In previous studies serum levels of miR-192 were associated with myocardial fibrosis^61^ as well as the development of ischemic heart failure in AMI patients^62^. This limitation, and - compared to biomarker studies beyond the miRNA research field - the relatively small patient groups analyzed in this work, calls for further validation in a larger and population-based prospective cohort study. We can neither exclude that our initial miRNA profiling study overlooked miRNAs that are potential biomarkers of diabetes and thereby escaped our validation studies. Thus, we identified only one of the miRNAs previoiusly associated with incident T2DM, namely miR-122^18^. In this respect it is noteworthy to emphasize the different samples and methods used by us and Willeit and colleagues^23^, namely exclusively serum versus plasma and serum, Exiqon versus Life Technologies, normalization to endogenous reference miRNAs versus artificial spike-in RNAs. In this respect it is worth mentioning that one of the reference miRNAs recommended by Exiqon, miR-103, has been associated with T2DM^22^ and NAFLD^63^. However, in our population, miR-103 was not associated with present or incident T2DM. Moreover, exclusion of miR-103 as a reference miRNA and hence normalization for miR-106a and miR-425 only did not change the results (not shown).

Neither did we identify any of the seventeen miRNAs that according to two recent meta-analysis of profiling studies on blood samples (i.e. plasma, serum, peripheral blood mononuclear cells, or whole blood) are associated with prevalent T2DM^13^. Among them, in the discovery study we observed a significant association of the most frequently T2DM-associated miRNA - miR-29a - with incident T2DM in the 2-year follow-up samples (P = 0.014) but not beyond. Because miR-29a did not fulfill one of our pre-defined inclusion criteria – consistent deregulation during the follow-up – we excluded it from the validation studies. Our discovery study may also have overlooked some of the seventeen miRNAs previously associated with present T2DM because we searched for miRNAs associated with incident T2DM rather than present T2DM. Circulating miRNAs may remain unchanged in patients with incident T2DM but alter only after onset of the disease.

In conclusion, we identified and validated increased serum levels of miR-192 and miR-194 as biomarkers of both prevalent and incident T2DM in the VIVIT cohort. The independence of their association from established risk factors and their ability to improve risk prediction in combination with established risk factors may make them useful for improving risk stratification of pre-diabetic patients. In this regard, the associations of serum and tissue miR-192 or miR-194 with NASH and diabetic nephropathy make them interesting for early control not only of euglycemia but also the prevention of diabetic complications. However, further studies are needed to test whether the results observed in the VIVIT cohort are replicated in other populations.

## Methods

### Study design

#### Circulating miRNAs were investigated in a clinical *discovery* study and *three clinical validation studies and* two *mouse models of diabetes*

For the initial discovery study as well as two clinical validation studies, 256 patients were selected from the larger VIVIT cohort^61^ based on the availability of fasting and non-hemolytic sera stored at −80 °C as well as enrollment and follow-up data for incident T2DM. In the *discovery step* we applied PCR-based arrays covering 372 miRNAs (Exiqon, Vedbaek, Denmark) to sera that were collected at baseline as well as at the 2-, 4- and 6-year follow-up visits from nine metabolic syndrome patients *with* incident T2DM and nine sex- and age-matched metabolic syndrome patients *without* incident T2DM (Supplemental Table S1). Candidate miRNAs were validated by using qRT-PCR. In the *first cross-sectional validation study we* examined the association of the candidate miRNAs with glycemic stages in sex- and age-matched patient groups: 43 patients with NFG, 43 patients with IFG, and 43 patients with manifest T2DM (Supplemental Table S3). In the *longitudinal validation study* we investigated the association of the candidate miRNAs with incident T2DM in 213 patients. Of these patients, 35 individuals developed T2DM during the 6-year observation period and 178 individuals did not (Supplemental Table S7).

In the Manchester follow-up study, levels of miR-192, miR-194 and miR-215 in sera from 59 patients with T1DM and 54 probands with NFG were evaluated using qRT-PCR.

Finally, the candidate miRNAs were compared between Akt2 knockout mice and wild type mice, as well as between the apoE3Leiden*CETP transgenic mice fed with a high fat diet versus low fat diet.

### VIVIT cohort

Patients were selected from a prospective cohort study enrolled by the Vorarlberg Institute for Vascular Investigation and Treatment (VIVIT; Feldkirch, Austria)^64^ between September 1999 and October 2000 while they underwent coronary angiography for the evaluation of either suspected or established stable CAD. Clinical and laboratory examinations were performed at enrollment and every two years thereafter during an 8-year follow-up period. The study was approved by the Ethics Committee of the University of Innsbruck (Austria) and was performed in accordance with relevant guidelines and regulations. All participants gave written informed consent.

T2DM was diagnosed by either a physician at VIVIT or a referring physician based on oGTT according to the ADA criteria^65^. Individuals were classified to have incident T2DM, if they had no T2DM at the baseline visit of the present investigation (which corresponds to the 2-year visit of the VIVIT study cohort published previously^64^), but developed T2DM at any time during subsequent six years of observation. Metabolic syndrome was diagnosed according to National Cholesterol Education Program-Adult Treatment Panel III (NCEP ATP III) criteria^66^. NFG was defined as fasting glucose levels <5.6 mmol/L (100 mg/dL) at *all* follow-up visits. IFG was defined as glucose levels between 5.6 and 6.9 mmol/L (100–125 mg/dL) at least at two follow-up visits.

### Manchester cohort

Recruitment of patients and collection of blood samples were conducted in hospitals of the Central Manchester University Hospitals NHS Foundation Trust. Patients between 18 and 75 years of age were enrolled. T1DM patients and euglycemic control subjects with a history or clinical and/or ECG evidence of CHD were excluded. The study was approved by the NRES Committee North West – Greater Manchester Central and was performed in accordance with relevant guidelines and regulations. All participants gave written informed consent.

### Animals

All experimental protocols were in accordance with institutional guidelines and were approved by the Greek Federal Veterinary Office. Akt2 knockout mice (Akt2−/−)^28^ and C57BL6/J wild type littermates were obtained from Dr Christos Tsatsanis (University of Crete Medical School). ApoE3Leiden and hCETP transgenic mice (E3L*CETP)^27^ mice were obtained from TNO Leiden. All mice were maintained at the animal facility of the Institute of Molecular Biology and Biotechnology of Heraklion Greece under pathogen free conditions on a 12 h light/dark cycle. Akt2−/− mice and their control littermates (n = 7 in each group) were fed standard rodent chow and sacrified at the age of 12 weeks. Starting at the age of 12 weeks male E3L*CETP mice were fed for 12 weeks with either a low fat diet containing 20% protein, 70% carbohydrate and 10% fat (LFD, n = 4) or a high fat diet containing 20% protein, 20% carbohydrate, 60% fat and 0,25% cholesterol (HFD, n = 6) before they were sacrificed. The mice were anesthetized with isoflurane and their blood was collected by cardiac puncture.

### Measurement of miRNAs in human serum

Total RNA was isolated from 200 µl serum using column-based systems (discovery sample: miRNeasy Mini Kit, Qiagen, Hilden, Germany; validation samples: miRCURY RNA Isolation Kit-Biofluids, Exiqon) according to the manufacturers’ protocols with minor modifications (see Supplemental Methods).The RNA was either immediately reverse transcribed or stored at −80 °C.

For the *discovery study*, 4 µL RNA solutions were reverse transcribed using the miRCURY LNA Universal RT cDNA synthesis kit (Exiqon). Samples were screened for the expression of 372 miRNAs by qRT-PCR based arrays (Human panel I V2.M, miRCURY LNA SYBR Green Master Mix; Exiqon) on a 7900HT Fast qRT-PCR System (Applied Biosystems, Foster City, California, USA). The amplification curves were examined with the SDS 2.4 software (Applied Biosystems). Normalized values (ΔCq) were obtained as the raw Cq value minus the arithmetic average of raw Cqs for all miRNAs with reliable results (Cq values < 37).

For the *validation studies*, 2 µL RNA solutions were reverse transcribed using the miRCURY LNA Universal RT cDNA synthesis kit (Exiqon). MiRNAs were measured using the ExiLENT SYBR Green Master Mix according to the manufacturer’s instructions. PCR assays were performed in triplicates on a LightCycler 480 II qRT-PCR System (Roche, Rotkreuz, Switzerland) and all miRNAs were detected at Cq < 37. The amplification curves were analyzed using the Light Cycler software version 1.5 (Roche). Normalized values (ΔCq) were obtained as the raw Cq value minus the arithmetic average of raw Cqs for the three reference miRNAs miR-103, miR-106a and miR-425. See Supplemental Methods for more details on normalization.

### Measurement of miRNAs in murine serum

The mice were anesthetized with isoflurane and their blood was collected by cardiac puncture. After 30 minutes at room temperature, blood samples were centrifuged twice at 3,000 rpms/4oC for 20 min each and the serum was used immediately or stored at −80 °C.

Isolation of total microRNAs from mice sera was performed using the miRCURY RNA Isolation Kit (Exiqon) according to manufacturer’s protocol. The analyzed serum volume was 50 μl for each mouse. Spike-in template (provided by the kit) was added (1 μL) in each serum sample and was used as reference. Isolated miRNA concentrations were measured in a TECAN Infinite 200® PRO instrument. Total miRNAs (20 ng) were subjected to cDNA synthesis by using the Universal cDNA Synthesis Kit II (Exiqon) as suggested by the manufacturer. The cDNAs were used for Real Time PCR analysis using the ExiLENT SYBR® Green master mix and the corresponding primer sets for each miRNA (Exicon) in an Applied Biosystems Step One Plus Real Time PCR system.

### Clinical chemistry

For the human studies, hemolysis indices as well as activities of AST, ALT, and GGT were determined by photometric tests on a cobas 8000 c701 modular analyzer (Roche). Serum insulin and C-peptide were determined by a chemiluminescence enzyme immunoassay on a LIAISON Analyzer (DiaSorin, Saluggia, Italy). Cystatine C was determined on a ProSpec nephelometer (Dade Behring, Switzerland). All other clinical chemistry parameters were measured in fresh samples as reported previously^64^.

Glucose in mouse blood was measured by using a GDH-based glucose measurement device (TRUEresult, Nipro-Medical Europe, Belgium). Serum insulin levels in mice were determined using enzymatic immunoassay (Mercodia Mouse Insulin ELISA, 10-1247-01.

### Statistical analysis

Differences in sample characteristics were tested using Chi-square tests for categorical variables and either the Mann-Whitney U Test or the Kruskal-Wallis Test for continuous variables. To compare miRNA levels between patient groups the Kruskal-Wallis test was run followed by Dunn’s procedure for pairwise comparisons and a Bonferroni correction for multiple testing. Correlations between variables were calculated using Spearman’s Rank correlation test. Prospective analysis for future T2DM was performed using binary logistic regression. For this, continuous variables were log transformed if necessary and standardized to have the same mean and SD. ORs of the miRNAs were calculated with −ΔCt values, because ΔCt values have an inverse correlation to increasing miRNA concentrations. Receiver operating characteristic curve analyses were used to determine the AUCs for established biomarkers, miRNAs and combinations thereof^67^. The optimum cutoff points of the AUCs to discriminate between „incident T2DM “ and „no incident T2DM “ were calculated by means of the Youden index^25^. Kaplan-Meier survival analysis followed by a log rank test was conducted to investigate any association of miRNA levels with the time to T2DM diagnosis. To compare miRNA levels between groups of mice the Student’s t-test was performed.

Statistical analyses were performed with SPSS (Version 23; IBM, Zürich, Switzerland) and graphs were created with Prism7 (GraphPad Software, La Jolla, California). All tests were 2-sided and a *P*-value < 0.05 was considered statistically significant.

## Electronic supplementary material

Supplementary Information
