# Supplementary material for: Circulating microRNAs -192 and -194 are associated with the presence and incidence of diabetes mellitus
Source: Sci Rep. 2018 Sep 24;8:14274. doi: 10.1038/s41598-018-32274-9 (PMC6155281; doi:10.1038/s41598-018-32274-9)
Supplement: Supplementary file 1 — Supplementary Information [file 41598_2018_32274_MOESM1_ESM.docx]

**Circulating microRNAs -192 and -194 are associated with the presence and incidence of diabetes mellitus**

Andrea Jaeger MSc ^1,2^, Lukas Zollinger BSc, Christoph H. Saely MD ^3,4,5^,

Axel Muendlein PhD^3^, Ioannis Evangelakos MSc^6^, Dimitris Nasias MSc^6^, Nikoleta Charizopoulou PhD^6^, Jonathan D. Schofield MD^7,8^,

Alaa Othman MD PhD , Handrean Soran MD^7,8^ , Dimitris Kardassis PhD^6^,

Heinz Drexel MD ^3,4,5,9^, and Arnold von Eckardstein MD^1,2*^

**Supplementary Information**

**Measurement of miRNAs in human serum**

Total RNA was isolated from 200 µl serum using column-based systems (discovery sample: miRNeasy Mini Kit, Qiagen, Hilden, Germany; validation samples: miRCURY RNA Isolation Kit-Biofluids, Exiqon) according to the manufacturers’ protocols with minor modifications: Before isolation, 1 µl of bacteriophage MS2 RNA (Roche) and 1 µl of 10 U/µl glycogen, RNA grade, (Thermo Fisher Scientific, Reinach, Switzerland) were added to increase the RNA yield. For the validation studies, a no-template control of water was purified with every batch of 12 samples and analyzed like the samples. For monitoring the isolation yield and absence of PCR inhibitors, an RNA spike-in was added and its recovery was tested via PCR (UniSp6; Exiqon). Phase Lock Gel Heavy Tubes (5 Prime, Hamburg, Germany) were used to improve phase separation and yield of nucleic acids. The RNA was either immediately reverse transcribed or stored at -80 °C.

For reverse transcription, Exiqon protocol recommends using RNA amounts based on starting volume rather than based on concentration. The typically low miRNA concentration in human sera and the carrier RNA, which was added during extraction, make optical measurements inappropriate for the assessment of RNA yield and quality. Hence, for the *discovery study*, 4 µL RNA solution were reverse transcribed in 20 µl reactions using the miRCURY LNA Universal RT cDNA synthesis kit (Exiqon). Then, cDNA was diluted 1:50 and assayed in 10 µL PCR reactions. Samples were screened for the expression of 372 miRNAs by qRT-PCR based arrays (Human panel I V2.M, miRCURY LNA SYBR Green Master Mix; Exiqon). All amplifications were performed in a 7900HT Fast qRT-PCR System (Applied Biosystems, Foster City, California, USA) in 384 well plates. The samples were included into analysis, if the RNA spike-in added during RNA extraction and two miRNAs typically detected in serum (miR-103 and miR-192) were detected at the expected levels. The amplification curves were examined with the SDS 2.4 software (Applied Biosystems). Normalized values (ΔCq) were obtained as the raw Cq value minus the arithmetic average of raw Cqs for all miRNAs with reliable results (Cq values <37).

For the *validation* *studies*, 2 µL (see explanation above) RNA solution were reverse transcribed in 10 µl reactions using the miRCURY LNA Universal RT cDNA synthesis kit (Exiqon). The cDNA was diluted 1:50 and was assayed in 10 µL PCR reactions. MiRNAs were measured using the ExiLENT SYBR Green Master Mix according to the manufacturer’s instructions. PCR assays were performed in triplicates on a LightCycler 480 II qRT-PCR System (Roche, Rotkreuz, Switzerland) and all miRNAs were detected at Cq<37. The amplification curves were analyzed using the Light Cycler software version 1.5 (Roche). The samples were included into analysis, if the RNA spike-in added during RNA extraction was detected at the expected level, all miRNAs were detected with 5 Cqs below the corresponding no-template control and the endogenous reference miRNAs (see next section) were detected at Cq<37.

For normalization, GeNorm 0 and NormFinder (2) algorithms identified miR-103, miR-106a and miR-425 as endogenous reference miRNAs. These miRNAs were previously used as reference genes [miR-106a (3); miR-103 (4)-(6); miR-425 (6)-(8)], proposed by the provider (Exiqon) and we did confirm their suitability for our study population (Supplemental Figure S1). Normalized values (ΔCq) were obtained as the raw Cq value minus the arithmetic average of raw Cqs for the three reference miRNAs. Utilizing the average of three reference miRNAs makes the analyses robust, since the possible bias of one reference miRNA is compensated by the others. The average of the selected reference miRNAs showed a highly significant correlation with the geometric mean of all detectable miRNAs (Pearson R = 0.978, p=1.96 E-54, Supplemental Figure S5). This suggests that selected miRNAs were sufficient and appropriate for the validation studies.

**Supplementary** **Figures**


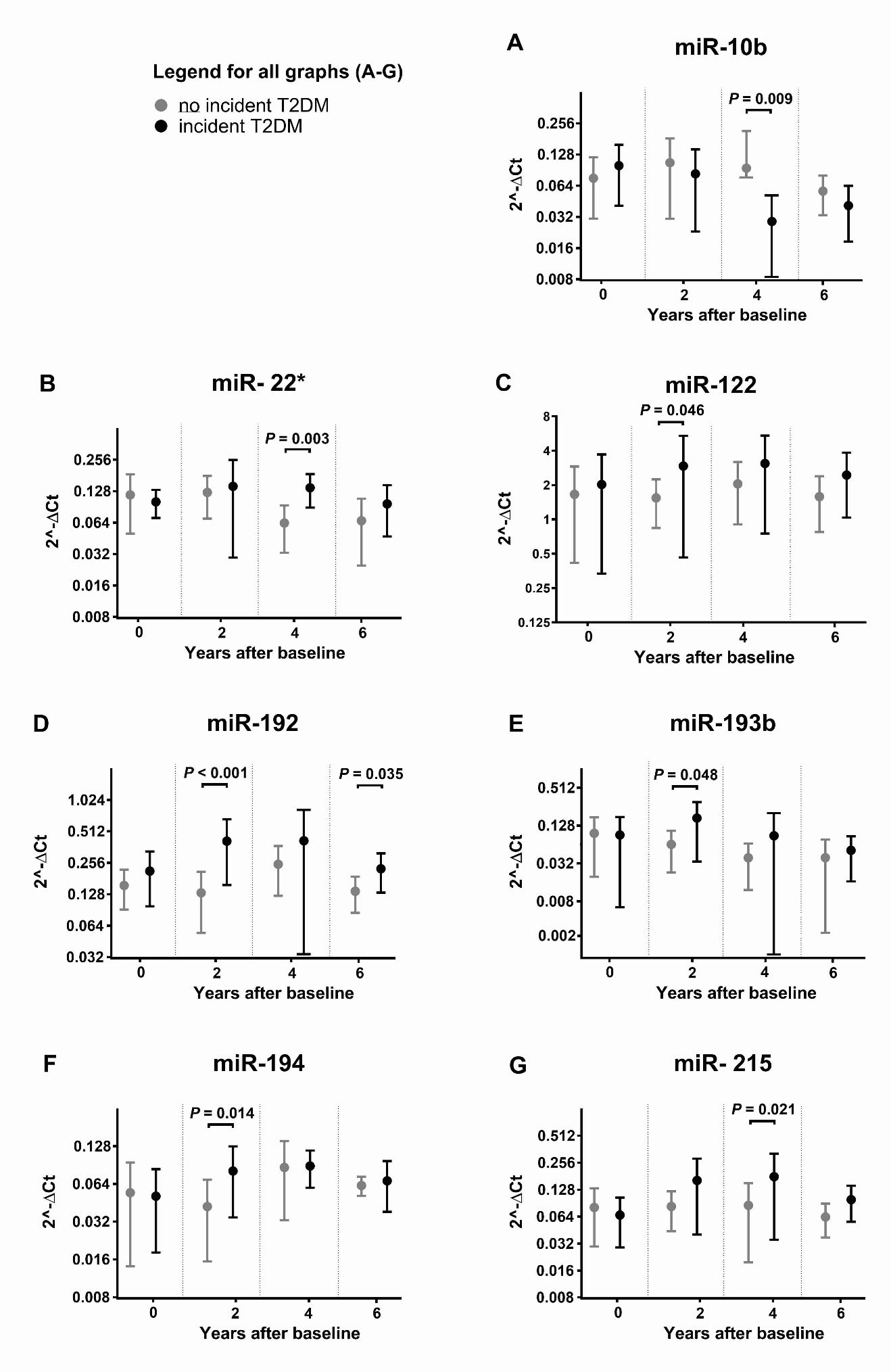


**Supplemental Figure S1: Identification of seven candidate miRNAs whose serum levels differ significantly between nine subjects with incident diabetes and nine subjects without incident diabetes at least once during 6 years of follow-up.** All 18 probands had metabolic syndrome but were free of manifest diabetes at baseline. Nine probands with incident diabetes were matched by age and sex with nine probands who did not develop diabetes. Blood samples for sera were collected every 2 years during follow-up. MiRNAs in sera were quantified by RT-PCR using an array from Exiqon encompassing 384 miRNAs. Values represent 2^−ΔCq^ values. Mean ± standard deviation is shown. Cq values have been normalized by the mean expression of all miRNAs detectable (Cq < 37) in all serum samples. Statistically significant differences were determined by the unpaired, two-sided t-test.


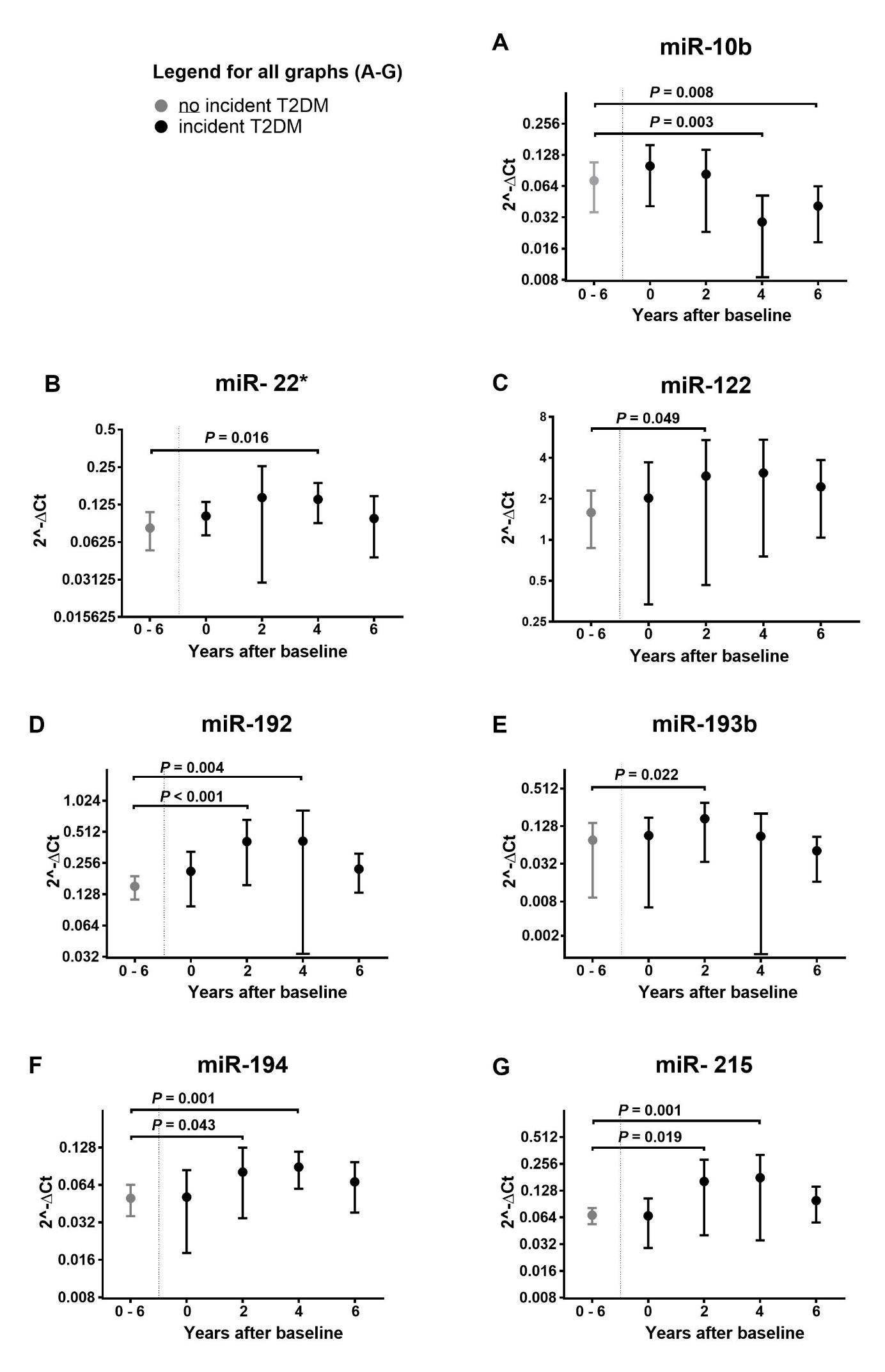


**Supplemental Figure S2:** Identification of seven candidate miRNAs whose serum levels differ significantly between nine subjects with incident diabetes and nine subjects without incident diabetes during 6 years of follow-up. The individual time points (shown in black) were statistically different from an aggregated value from all of the non-diabetic time points (shown in grey). P-values were calculated with on-way ANOVA.


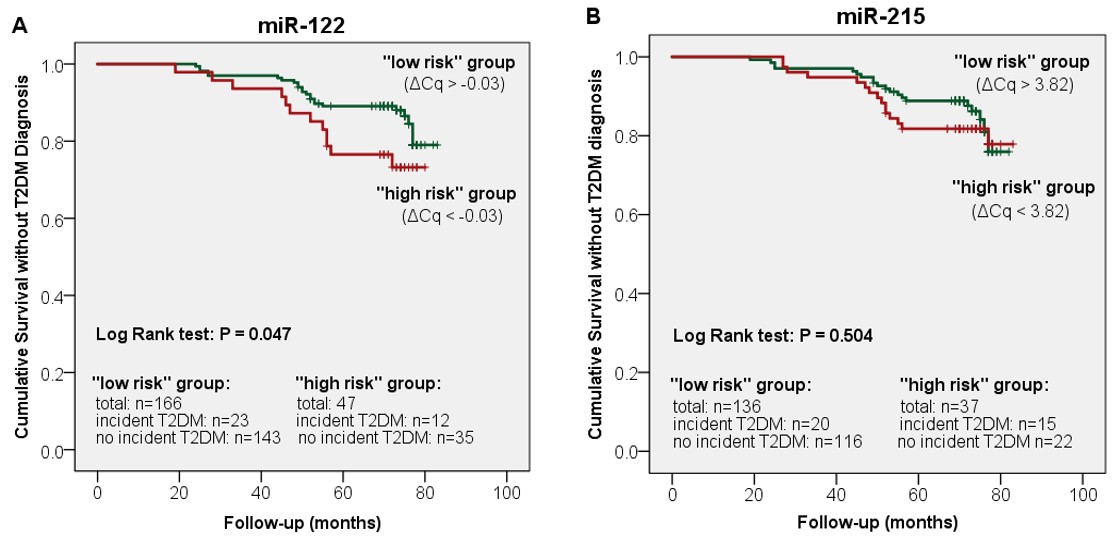


**Supplemental Figure S3: Time to diabetes-diagnosis for patients with low versus elevated miR-122 or miR-215 levels.** Kaplan-Meier survival analysis shows significantly different survival distributions for «low risk» patients with low levels of circulating miR-122 (A) versus «high risk» patients with elevated miRNA concentrations (log rank test, *P* = 0.047). There was no significant difference in survival distribution for different miR-215 concentrations (B). Patients were assigned to either a „low risk“ or a „high risk“ group for future diabetes depending on whether their miRNA levels were below or above the optimum cutoff points calculated with the Youden-Index (-0.03 ΔCq for miR-122, and 3.82 ΔCq for miR-215).

**Supplemental Figure S4: Serum insulin levels of AKT knockout mice vs non diabetic controls (a) and apoE3Leiden*CETP transgenic mice fed with low fat diet or with high fat diet (b).** Akt -/- mice: Akt knockout mice, E3L*CETP mice: apoE3Leiden*CETP transgenic mice, HFD: high fat diet, LFD: low fat diet


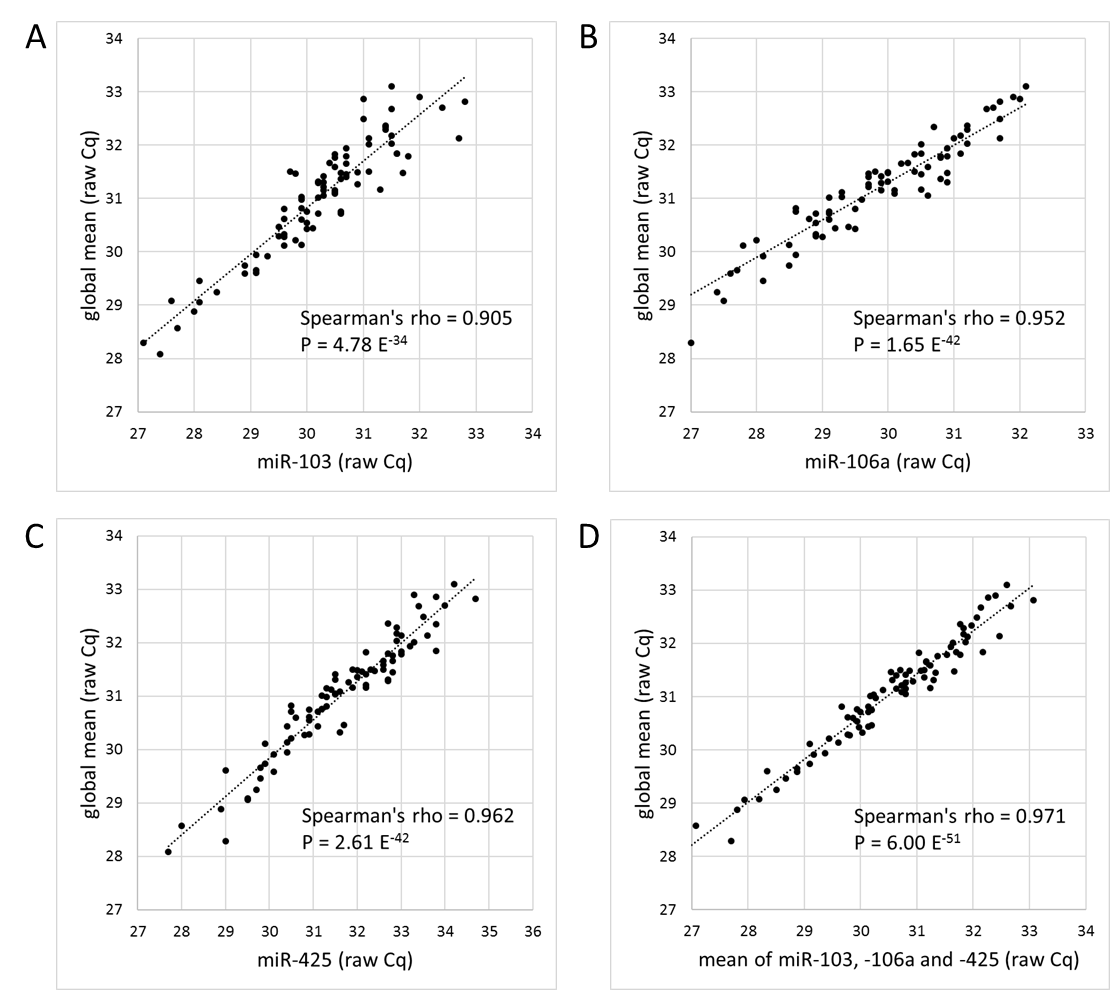


**Supplemental Figure S5: Assessing the suitability of the selected reference miRNAs for normalization in the validation studies.** Spearman correlations between the global mean and miR-103 (A), miR-106a (B), miR-425 (C) as well as the mean of the selected reference miRNAs (D) are shown in the discovery sample (n=72). The global mean was used for normalization in the discovery study and the mean of miR-103, miR-106a and miR-425 was used for normalization in the validation studies. The geometric mean of the selected reference miRNAs (raw Cq) showed a highly significant correlation with the geometric mean of all detectable miRNAs (Pearson *R* = 0.978, *p*=1.96 E^-54^). This suggests that selected miRNAs were sufficient and appropriate for the validation studies. Global mean: Mean expression of all miRNAs detectable in all samples (Ct<37)

**Supplementary Tables**

**Supplemental Table S1:** **Baseline** **patient characteristics of the VIVIT discovery study cohort.**

|  | **no incident diabetes (n=9)** | | **incident diabetes (n=9)** | | ***P*-value** |
| --- | --- | --- | --- | --- | --- |
| Sex (female) | 1 | (11%) | 1 | (11%) | 1.000 |
| Age [years] | 60 | (49-64) | 54 | (50-64) | 0.931 |
| MetS (ATPIII) | 9 | (100%) | 9 | (100%) | 1.000 |
| BMI | 30.4 | (28.4-32.2) | 29.7 | (27.1-31.0) | 0.605 |
| Fasting glucose [mmol/l] | 5.9 | (5.7-6.3) | 5.9 | (5.5-6.7) | 0.730 |
| HbA1c [%] ^a)^ | 5.7 | (5.6-6.1) | 6.0 | (5.8-6.1) | 0.436 |
| Significant coronary stenosis ≥50% | 5 | (56%) | 6 | (67%) | 0.629 |
| Myocardial infarction | 4 | (44%) | 2 | (22%) | 0.317 |
| Stroke | 0 | (0%) | 1 | (11%) | 0.303 |

Continuous variables are presented as medians with interquartile ranges; Categorical variables are presented as numbers and percentages. *P*-values were calculated using the Mann-Whitney U test for continuous variables and the χ^2^ test for categorical variables. ^a)^ To convert % HbA1c to mmol/mol, multiply by 10.9 and subtract 23.5.

|  |  | miR-10b | | | | miR-22* | | | | miR-122 | | | | miR-192 | | | |
| --- | --- | --- | --- | --- | --- | --- | --- | --- | --- | --- | --- | --- | --- | --- | --- | --- | --- |
| Patient ID | incident diabetes  (0=no, 1=yes) | 0 y | 2 y | 4 y | 6 y | 0 y | 2 y | 4 y | 6 y | 0 y | 2 y | 4 y | 6 y | 0 y | 2 y | 4 y | 6 y |
| 52 | 0 | 3.88 | 3.14 | 5.09 | 4.46 | 2.48 | 2.64 | 4.19 | 4.26 | -2.12 | -1.33 | -0.40 | -0.94 | 1.78 | 3.94 | 2.49 | 2.16 |
| 86 | 0 | 2.77 | 3.53 | 3.24 | 4.27 | 4.27 | 3.33 | 3.64 | 4.17 | -0.33 | 0.43 | -1.54 | -0.03 | 3.27 | 3.13 | 2.34 | 3.07 |
| 185 | 0 | 5.06 | 2.07 | 4.28 | 3.87 | 2.96 | 2.87 | 3.78 | 4.57 | 1.16 | 1.17 | -1.10 | 0.07 | 2.36 | 1.77 | 2.18 | 2.87 |
| 207 | 0 | 4.01 | 3.89 | 4.19 | 4.51 | 3.81 | 2.49 | 3.99 | 3.71 | 1.01 | -0.41 | 0.10 | 0.61 | 2.91 | 2.09 | 2.09 | 3.31 |
| 320 | 0 | 4.11 | 5.02 | 4.59 | 4.32 | 2.51 | 3.42 | 4.39 | 3.92 | -0.79 | -0.08 | 0.01 | -1.18 | 2.51 | 3.32 | 2.79 | 2.72 |
| 378 | 0 | 5.32 | 3.81 | 4.29 | 5.52 | 3.02 | 2.91 | 2.99 | 2.62 | -0.98 | -0.89 | -1.90 | 0.12 | 2.22 | 3.21 | 0.89 | 2.72 |
| 396 | 0 | 4.04 | 4.04 | 4.90 | 3.30 | 2.14 | 6.54 | 4.90 | 5.70 | -0.76 | -0.76 | -0.48 | -0.40 | 3.44 | 3.14 | 1.70 | 3.10 |
| 592 | 0 | 3.06 | 2.12 | 1.30 | 4.34 | 5.06 | 2.42 | 5.30 | 3.44 | -0.74 | -0.98 | -0.88 | -1.06 | 2.76 | 3.72 | 2.90 | 4.84 |
| 797 | 0 | 3.75 | 4.11 | 3.68 | 3.67 | 4.35 | 3.11 | 3.78 | 5.27 | -1.35 | -1.19 | -2.10 | -1.63 | 3.25 | 3.61 | 1.88 | 2.37 |
| 5 | 1 | 2.44 | 3.60 | 7.18 | 7.38 | 3.04 | 1.70 | 2.38 | 2.88 | 0.04 | -1.20 | -2.00 | -1.32 | 3.34 | 1.50 | 0.88 | 1.98 |
| 14 | 1 | 2.95 | 4.09 | 4.64 | 4.78 | 2.65 | 3.69 | 2.44 | 2.68 | -0.15 | -1.71 | -2.04 | -0.42 | 1.55 | 1.39 | 2.04 | 3.28 |
| 32 | 1 | 4.71 | 7.72 | 5.72 | 5.71 | 3.91 | 3.72 | 3.02 | 4.51 | 0.01 | 0.02 | -0.87 | -0.59 | 2.81 | 1.72 | 2.02 | 2.01 |
| 45 | 1 | 4.24 | 3.21 | 6.12 | 4.81 | 3.14 | 4.21 | 4.42 | 4.21 | -0.76 | -1.89 | -1.27 | -2.39 | 2.94 | 1.31 | 1.92 | 1.41 |
| 138 | 1 | 4.44 | 3.99 | 5.49 | 4.56 | 3.14 | 2.79 | 2.29 | 3.86 | -1.16 | -1.21 | -1.89 | -0.94 | 1.44 | 1.49 | 2.59 | 2.36 |
| 148 | 1 | 4.47 | 2.39 | 6.04 | 4.05 | 3.47 | 3.79 | 3.34 | 2.55 | -0.33 | -1.46 | -0.25 | -0.06 | 2.17 | 2.59 | 1.94 | 2.94 |
| 184 | 1 | 3.23 | 2.87 | 4.76 | 3.69 | 4.03 | 1.47 | 2.66 | 3.99 | -2.67 | -3.13 | -3.13 | -1.81 | 1.43 | -0.03 | -0.54 | 2.39 |
| 362 | 1 | 3.23 | 3.35 | 3.65 | 4.16 | 3.63 | 3.45 | 3.25 | 3.76 | -0.57 | 0.05 | -1.83 | -0.44 | 4.63 | 2.45 | 1.95 | 2.46 |
| 793 | 1 | 2.42 | 6.16 | 5.73 | 4.05 | 3.12 | 3.26 | 2.93 | 2.55 | -1.08 | -1.84 | 0.44 | -1.85 | 3.22 | 0.86 | 1.73 | 1.55 |

**Supplemental Table S2: Normalized Cq values (ΔCqs) of the candidate miRNAs measured in the VIVIT discovery study.**

**Supplemental Table S2 (continued): Normalized Cq values (ΔCqs) of the candidate miRNAs measured in the VIVIT discovery study.**

|  |  | miR-193b | | | | miR-194 | | | | miR-215 | | | |
| --- | --- | --- | --- | --- | --- | --- | --- | --- | --- | --- | --- | --- | --- |
| Patient ID | incident diabeetes  (0=no, 1=yes) | 0 y | 2 y | 4 y | 6 y | 0 y | 2 y | 4 y | 6 y | 0 y | 2 y | 4 y | 6 y |
| 52 | 0 | 5.18 | 4.44 | 8.06 | 5.19 | 3.38 | 6.34 | 4.39 | 4.16 | 2.78 | 3.74 | 3.79 | 4.16 |
| 86 | 0 | 3.67 | 7.53 | 4.77 | 4.04 | 2.97 | 6.03 | 4.14 | 3.97 | 2.57 | 4.23 | 3.64 | 4.17 |
| 185 | 0 | 3.56 | 5.87 | 7.87 | 7.88 | 6.06 | 4.37 | 3.38 | 4.17 | 6.06 | 2.87 | 3.78 | 4.07 |
| 207 | 0 | 3.31 | 3.59 | 3.91 | 3.99 | 4.11 | 5.59 | 4.19 | 4.51 | 3.91 | 4.19 | 3.29 | 4.31 |
| 320 | 0 | 1.91 | 2.82 | 5.42 | 6.09 | 4.21 | 3.52 | 3.09 | 4.12 | 3.31 | 3.82 | 4.09 | 3.42 |
| 378 | 0 | 5.32 | 4.21 | 5.02 | 3.49 | 5.32 | 4.41 | 2.29 | 4.02 | 5.32 | 3.61 | 1.99 | 4.12 |
| 396 | 0 | 4.64 | 3.94 | 7.40 | 4.90 | 6.34 | 3.74 | 4.90 | 3.90 | 3.64 | 4.14 | 4.90 | 6.20 |
| 592 | 0 | 5.26 | 3.52 | 3.24 | 5.30 | 4.06 | 4.41 | 3.70 | 3.94 | 3.46 | 2.62 | 5.30 | 3.64 |
| 797 | 0 | 2.55 | 3.71 | 3.67 | 4.88 | 5.25 | 5.71 | 3.68 | 3.67 | 4.15 | 3.91 | 3.58 | 3.27 |
| 5 | 1 | 2.34 | 2.80 | 3.78 | 3.78 | 5.14 | 4.00 | 2.98 | 3.68 | 5.14 | 2.60 | 2.48 | 3.58 |
| 14 | 1 | 3.55 | 3.89 | 6.88 | 3.84 | 4.85 | 3.49 | 3.24 | 3.98 | 3.25 | 2.89 | 3.54 | 3.28 |
| 32 | 1 | 6.51 | 4.02 | 4.51 | 5.82 | 4.81 | 4.52 | 4.22 | 4.41 | 5.01 | 4.62 | 3.32 | 2.61 |
| 45 | 1 | 6.64 | 3.11 | 3.11 | 4.62 | 5.14 | 3.21 | 3.52 | 3.01 | 6.64 | 2.01 | 2.72 | 3.11 |
| 138 | 1 | 4.04 | 2.59 | 3.76 | 4.09 | 3.44 | 4.09 | 3.19 | 4.16 | 3.14 | 3.69 | 3.09 | 3.96 |
| 148 | 1 | 5.97 | 4.39 | 7.25 | 6.04 | 5.27 | 3.69 | 4.24 | 5.34 | 3.97 | 3.99 | 3.54 | 4.34 |
| 184 | 1 | 2.03 | 0.97 | 3.79 | 1.46 | 3.03 | 2.37 | 3.96 | 3.29 | 3.53 | 1.17 | 0.86 | 2.69 |
| 362 | 1 | 3.43 | 4.00 | 4.46 | 2.65 | 4.93 | 4.35 | 3.45 | 4.36 | 3.33 | 2.85 | 2.45 | 4.46 |
| 793 | 1 | 3.52 | 2.26 | 7.25 | 6.33 | 4.02 | 4.26 | 3.13 | 3.75 | 4.02 | 2.76 | 2.73 | 3.25 |

By means of a PCR based screening platform (Exiqon) we analyzed sera of nine patients with metabolic syndrome who developed diabetes during a 6-year observation period (incident diabetes) and nine patients who did not (no incident diabetes). Circulating miR profiles were recorded in four sera per patient that were collected at baseline as well as at the 2-, 4-, and 6-year visits. The candidate miRs were normalized to the mean expression of all miRs with reliable result (Cq<37).

**Supplemental Table S3: Characteristics of the VIVIT cross-sectional validation study cohort**

|  | **NFG (n=43)** | | **IFG (n=43)** | | **diabetes (n=43)** | | **P-value** |
| --- | --- | --- | --- | --- | --- | --- | --- |
| Sex (female) | 11 | (26%) | 11 | (26%) | 11 | (26%) | 1.000 |
| Age [years] | 62 | (53-69) | 60 | (55-67) | 64 | (54-69) | 0.778 |
| **MetS (ATPIII)** | **4** | **(9%)** | **16** | **(37%)** | **33** | **(77%)** | **<0.001 *** |
| **BMI** | **25.6** | **(23.9-28.8)** | **28.7** | **(25.5-30.7)** | **28.7** | **(25.9-32.9)** | **0.003 ^†^** |
| **Fasting glucose [mmol/l]** | **4.8** | **(4.7-5.2)** | **5.8** | **(5.6-6.0)** | **9.5** | **(7.4-10.9)** | **<0.001 *** |
| **HbA1c [%]** ^‡^ | **5.5** | **(5.2-5.7** | **5.7** | **(5.5-6.1)** | **7.3** | **(6.5-8.4)** | **<0.001 *** |
| **C-peptide [µg/l]** | **1.8** | **(1.2-2.2)** | **2.5** | **(1.9-3.3)** | **2.9** | **(2.1-3.7)** | **<0.001 ^†^** |
| **Insulin [mU/l]** | **5.5** | **(3.9-8.9)** | **10.2** | **(6.4-14.9)** | **11.9** | **(7.2-19.5)** | **<0.001 ^†^** |
| Creatinine [µmol/l] | 89 | (71-97) | 89 | (80-97) | 89 | (71-89) | 0.288 |
| eGFR (CKD-EPI) [ml/min/1.73 m^2^] | 77 | (67-89) | 74 | (68-87) | 83 | (65-90) | 0.466 |
| **Significant coronary stenosis ≥50%** | **18** | **(42%)** | **28** | **(65%)** | **28** | **(65%)** | **0.042** |
| Myocardial infarction | 10 | (23%) | 17 | (40%) | 19 | (44%) | 0.104 |
| Stroke | 3 | (7%) | 0 | (0%) | 5 | (12%) | 0.079 |

Continuous variables are presented as medians with interquartile ranges; Categorical variables are presented as numbers and percentages. P-values were calculated using the Kruskal-Wallis test for continuous variables and the χ2 test for categorical variables. Parameters with significant differences are shown in bold font. † Represents a significant difference between NFG and the other two patient groups. * Represents a significant difference between all three patient groups. ‡ To convert %HbA1c to mmol/mol, multiply by 10.9 and subtract 23.5

| Patient ID | glycemic stage  (0=NFG, 1=IFG, 2=T2DM) | miR-122 (ΔCq) | miR-192 (ΔCq) | miR-194 (ΔCq) | miR-215 (ΔCq) |
| --- | --- | --- | --- | --- | --- |
| 37 | 2 | 0.83 | 2.41 | 3.39 | 3.59 |
| 50 | 2 | 0.68 | 1.57 | 3.19 | 4.45 |
| 60 | 2 | -1.11 | 1.69 | 1.45 | 2.22 |
| 69 | 2 | -1.69 | 1.15 | 1.66 | 3.33 |
| 92 | 2 | 1.98 | 2.20 | 2.79 | 4.21 |
| 95 | 2 | 2.25 | 2.12 | 4.05 | 2.78 |
| 98 | 2 | -0.13 | 1.99 | 2.38 | 3.94 |
| 135 | 2 | -2.94 | -0.14 | 0.26 | 1.03 |
| 143 | 2 | 2.16 | 2.37 | 3.37 | 4.25 |
| 152 | 2 | 1.65 | 1.92 | 3.01 | 3.16 |
| 163 | 2 | 0.73 | 1.13 | 2.58 | 2.79 |
| 211 | 2 | 2.08 | 2.62 | 3.59 | 4.15 |
| 213 | 2 | 2.08 | 0.80 | 2.55 | 2.99 |
| 224 | 2 | 0.37 | 1.65 | 2.83 | 3.12 |
| 294 | 2 | 2.15 | 1.50 | 1.84 | 4.28 |
| 295 | 2 | 0.81 | 0.70 | 2.30 | 2.14 |
| 332 | 2 | 2.47 | 2.54 | 2.62 | 4.20 |
| 337 | 2 | 1.92 | 2.70 | 3.28 | 4.35 |
| 347 | 2 | -1.24 | -0.80 | 0.36 | 3.02 |
| 384 | 2 | -0.71 | 0.55 | 1.62 | 4.38 |
| 386 | 2 | -1.10 | -0.71 | 1.12 | 3.06 |
| 395 | 2 | -0.03 | 0.09 | 3.19 | 4.31 |
| 402 | 2 | 1.55 | 3.27 | 3.48 | 4.51 |
| 407 | 2 | 1.15 | 1.92 | 3.63 | 3.75 |
| 417 | 2 | 2.22 | 3.52 | 3.37 | 5.72 |
| 418 | 2 | -0.21 | 1.99 | 1.82 | 3.05 |
| 435 | 2 | 1.64 | 0.01 | 0.05 | 4.42 |
| 473 | 2 | 0.52 | 1.21 | 3.26 | 4.02 |
| 480 | 2 | 0.67 | 1.44 | 2.75 | 3.58 |
| 481 | 2 | -2.33 | -1.19 | 1.32 | 2.84 |
| 522 | 2 | -0.38 | -0.59 | 0.90 | 3.23 |
| 620 | 2 | 3.22 | 3.44 | 3.92 | 5.16 |
| 675 | 2 | -1.51 | 2.25 | 2.23 | 3.96 |
| 693 | 2 | -0.40 | 0.81 | 1.29 | 3.01 |
| 706 | 2 | 0.60 | 1.91 | 2.78 | 3.81 |
| 721 | 2 | 1.81 | 1.73 | 2.08 | 4.67 |
| 751 | 2 | 1.25 | 1.65 | 2.71 | 3.36 |
| 755 | 2 | 1.77 | 3.16 | 2.53 | 4.70 |

**Supplemental Table S4:** **Normalized Cq values (ΔCqs) of the candidate miRNAs investigated for the association with glycemic stages in the VIVIT cohort.**

**Supplemental Table S4 (continued a):** **Normalized Cq values (ΔCqs) of the candidate miRNAs investigated for the association with glycemic stages in the VIVIT cohort.**

| Patient ID | glycemic stage  (0=NFG, 1=IFG, 2=T2DM) | miR-122 (ΔCq) | miR-192 (ΔCq) | miR-194 (ΔCq) | miR-215 (ΔCq) |
| --- | --- | --- | --- | --- | --- |
| 758 | 2 | -0.83 | 1.22 | 2.34 | 3.13 |
| 766 | 2 | -0.47 | 1.46 | 2.29 | 3.12 |
| 772 | 2 | -1.02 | 1.27 | 1.06 | 2.62 |
| 789 | 2 | -1.41 | 0.42 | 1.22 | 2.58 |
| 799 | 2 | 1.07 | 3.08 | 2.78 | 4.25 |
| 185 | 1 | 0.97 | 2.91 | 3.04 | 4.88 |
| 207 | 1 | 2.57 | 2.21 | 3.32 | 4.26 |
| 396 | 1 | 1.30 | 1.90 | 3.17 | 5.16 |
| 592 | 1 | -0.64 | 1.38 | 2.11 | 4.26 |
| 797 | 1 | -0.26 | 1.96 | 2.90 | 4.76 |
| 25 | 1 | 2.11 | 1.90 | 3.16 | 4.86 |
| 53 | 1 | 0.65 | 2.97 | 1.86 | 4.10 |
| 80 | 1 | 2.07 | 3.11 | 3.93 | 4.53 |
| 119 | 1 | 1.26 | 1.03 | 3.65 | 4.62 |
| 132 | 1 | 0.24 | 2.23 | 3.99 | 2.53 |
| 164 | 1 | 1.43 | 2.79 | 5.37 | 4.34 |
| 179 | 1 | 0.63 | 1.45 | 4.46 | 3.71 |
| 194 | 1 | 4.41 | 3.49 | 6.56 | 5.19 |
| 229 | 1 | 0.60 | 2.96 | 3.43 | 3.67 |
| 235 | 1 | -1.50 | 1.69 | 1.28 | 3.20 |
| 237 | 1 | 0.00 | 1.65 | 2.85 | 3.86 |
| 259 | 1 | 1.14 | 3.11 | 3.35 | 4.46 |
| 272 | 1 | -0.15 | 1.51 | 2.55 | 2.78 |
| 305 | 1 | -1.98 | 1.40 | 1.88 | 3.08 |
| 331 | 1 | -0.42 | -0.33 | 0.60 | 3.96 |
| 340 | 1 | 0.23 | 1.92 | 2.57 | 4.70 |
| 355 | 1 | 0.72 | 2.99 | 3.66 | 3.70 |
| 397 | 1 | 2.07 | 2.44 | 3.15 | 4.26 |
| 414 | 1 | 1.35 | 0.80 | 1.94 | 4.87 |
| 433 | 1 | 1.90 | 3.51 | 3.32 | 4.07 |
| 436 | 1 | 1.46 | 3.17 | 3.97 | 4.44 |
| 462 | 1 | 3.18 | 3.25 | 4.47 | 5.16 |
| 527 | 1 | 0.87 | 2.16 | 3.45 | 3.54 |
| 530 | 1 | 2.07 | 2.04 | 3.15 | 4.54 |
| 593 | 1 | 1.73 | 3.36 | 3.43 | 4.79 |
| 596 | 1 | 0.38 | 2.78 | 3.41 | 4.61 |
| 601 | 1 | 0.29 | 2.34 | 2.89 | 4.90 |
| 673 | 1 | 0.71 | 1.58 | 2.72 | 3.89 |
| 691 | 1 | 1.56 | 2.37 | 2.38 | 4.67 |
| 698 | 1 | -0.10 | 1.87 | 2.59 | 4.20 |
| 741 | 1 | 1.77 | 1.71 | 2.43 | 4.17 |
| 764 | 1 | -1.96 | 1.19 | 1.39 | 2.78 |
| 782 | 1 | -2.38 | 0.16 | 1.13 | 1.99 |
| 788 | 1 | 0.70 | 3.13 | 3.34 | 4.17 |
| 125 | 1 | 1.32 | 2.55 | 2.85 | 4.33 |
| 210 | 1 | 0.78 | 2.49 | 3.22 | 4.36 |
| 44 | 1 | -1.10 | 1.33 | 1.97 | 2.88 |
| 690 | 1 | -0.67 | 1.42 | 1.90 | 4.20 |
| 21 | 0 | 2.36 | 2.82 | 3.26 | 4.50 |
|  |  |  |  |  |  |

**Supplemental Table S4 (continued b):** **Normalized Cq values (ΔCqs) of the candidate miRNAs investigated for the association with glycemic stages in the VIVIT cohort.**

| Patient ID | glycemic stage (0=NFG, 1=IFG, 2=T2DM) | miR-122 (ΔCq) | miR-192 (ΔCq) | miR-194 (ΔCq) | miR-215 (ΔCq) |
| --- | --- | --- | --- | --- | --- |
| 70 | 0 | 1.09 | 1.42 | 3.42 | 3.60 |
| 77 | 0 | 4.73 | 5.49 | 4.48 | 4.91 |
| 85 | 0 | -1.19 | 0.84 | 1.85 | 2.62 |
| 91 | 0 | 3.49 | 3.13 | 3.39 | 3.72 |
| 123 | 0 | -0.01 | 2.63 | 2.66 | 4.31 |
| 181 | 0 | 2.91 | 3.96 | 5.62 | 4.74 |
| 201 | 0 | -0.08 | 1.22 | 2.64 | 2.65 |
| 219 | 0 | 1.99 | 1.83 | 2.92 | 3.73 |
| 227 | 0 | -0.50 | 1.90 | 2.69 | 4.56 |
| 232 | 0 | -0.11 | 1.92 | 2.51 | 3.12 |
| 256 | 0 | 1.24 | 1.68 | 4.74 | 5.49 |
| 291 | 0 | 1.12 | 1.62 | 2.88 | 4.44 |
| 301 | 0 | 2.42 | 3.00 | 3.60 | 5.20 |
| 348 | 0 | 0.72 | 2.99 | 3.66 | 3.70 |
| 380 | 0 | 0.85 | 0.88 | 1.99 | 5.23 |
| 408 | 0 | 1.35 | 0.80 | 1.94 | 4.87 |
| 448 | 0 | 0.05 | 1.00 | 2.30 | 2.75 |
| 492 | 0 | 0.87 | 2.18 | 3.66 | 4.33 |
| 499 | 0 | -0.46 | 2.20 | 3.50 | 3.78 |
| 515 | 0 | 1.31 | 2.70 | 3.50 | 4.60 |
| 516 | 0 | 0.37 | 2.60 | 3.10 | 5.61 |
| 553 | 0 | 1.62 | 2.10 | 2.45 | 5.54 |
| 559 | 0 | 0.43 | 2.45 | 2.12 | 3.10 |
| 587 | 0 | -0.62 | 1.67 | 2.10 | 4.82 |
| 589 | 0 | 1.41 | 3.27 | 3.22 | 4.50 |
| 611 | 0 | 0.00 | 2.01 | 2.57 | 4.39 |
| 617 | 0 | 0.05 | 1.84 | 2.58 | 5.38 |
| 626 | 0 | 0.62 | 3.39 | 3.00 | 5.25 |
| 632 | 0 | -0.14 | 0.75 | 2.35 | 3.49 |
| 635 | 0 | 1.64 | 2.75 | 5.34 | 4.26 |
| 668 | 0 | 1.65 | 2.56 | 3.27 | 4.78 |
| 702 | 0 | 1.88 | 2.63 | 2.77 | 4.95 |
| 710 | 0 | 1.57 | 2.75 | 2.74 | 5.43 |
| 722 | 0 | 1.56 | 2.38 | 2.67 | 3.72 |
| 731 | 0 | 0.32 | 2.01 | 2.64 | 4.07 |
| 742 | 0 | 1.92 | 2.85 | 4.00 | 6.12 |
| 770 | 0 | 0.85 | 2.73 | 2.73 | 3.74 |
| 786 | 0 | 2.71 | 2.98 | 3.58 | 4.02 |
| 64 | 0 | 5.40 | 4.05 | 3.42 | 4.95 |
| 623 | 0 | 1.58 | 0.75 | 1.59 | 5.20 |
| 470 | 0 | 1.50 | 2.80 | 3.70 | 4.65 |
| 321 | 0 | 2.38 | 2.74 | 3.72 | 4.94 |

By means of individual qRT-PCR assays (Exiqon) we analyzed sera of 43 patients with NFG, 43 patients with IFG, and 43 patients with manifest diabetes. Circulating miRs were recorded at baseline. The candidate miRs were normalized to the mean expression of miR-103, miR-106a and miR-425. NFG, normal fasting glucose; IFG, impaired fasting glucose; T2DM, manifest type 2 diabetes mellitus.

**Supplemental Table S5: Characteristics of the Manchester study cohort**

|  | NFG | | T1DM | | *P*-value |
| --- | --- | --- | --- | --- | --- |
| Total | 53 | 100% | 54 | 100% | n.a. |
| Sex (female) | 37 | 69.8% | 29 | 53.7% | 0.087 |
| Age [years] | 40.0 | (35.0-50.0) | 38.5 | (30.5-47.3) | 0.212 |
| BMI | 25.39 | (22.22-27.66) | 25.36 | (22.95-28.91) | 0.501 |
| Glucose | 4.49 | (4.16-4.91) | 8.57 | (5.46-11.75) | 0.000 |
| eGFR | 97.55 | (83.03-108.73) | 106 | (92.42-117.20) | 0.044 |
| Total cholesterol [mmol/l] | 5.53 | (4.56-6.05) | 4.78 | (4.18-5.32) | 0.001 |
| LDL-cholesterol [mmol/l] | 3.34 | (2.80-3.80) | 2.72 | (2.20-3.10) | 0.000 |
| HDL-cholesterol [mmol/l] | 1.34 | (1.13-1.63) | 1.60 | (1.38-1.92) | 0.001 |
| Triglycerides [mmol/l] | 1.22 | (0.77-1.86) | 0.80 | (0.70-1.20) | 0.003 |
| Statins | 1 | 1.9% | 5 | 9.3% | 0.097 |
| Insulin | 0 | 0% | 54 | 100% | 0.000 |
| Metformin | 0 | 0% | 0 | 0% | n.a. |
| GLP1 related medication | 0 | 0% | 0 | 0% | n.a. |
| ACE inhibitors | 0 | 0% | 4 | 7.4% | 0.043 |
| Beta blockers | 0 | 0% | 1 | 1.9% | 0.320 |
| AT_1_-receptor antagonists | 2 | 3.8% | 2 | 3.7% | 0.985 |
| Acetylsalicylic acid | 0 | 0% | 1 | 1.9% | 0.320 |
| Calcium channel blockers | 2 | 3.8% | 0 | 0% | 0.150 |
| Cholecalciferol | 0 | 0% | 1 | 1.9% | 0.320 |

*Categorical variables are presented as numbers and percentages, continuous variables as medians with interquartile ranges. For continuous variables, the p-values were calculated using the Mann-Whitney U Test. The categorical variables were calculated using the Pearson’s chi-squared test.*

**Supplemental Table S6:** **Normalized Cq values (ΔCqs) of the candidate miRNAs investigated for the association with T1DM in the Manchester cohort.**

| Patient ID | incident T1DM  (0=no, 1=yes) | miR-192 (ΔCq) | miR-194 (ΔCq) | miR-215 (ΔCq) |
| --- | --- | --- | --- | --- |
| HS1 | 0 | 2.84 | 4.02 | 4.69 |
| HS2 | 0 | 2.96 | 5.24 | 4.55 |
| HS3 | 0 | 1.24 | 3.07 | 2.50 |
| HS4 | 0 | 3.08 | 3.60 | 3.51 |
| HS5 | 0 | 2.39 | 4.40 | 3.80 |
| HS7 | 0 | 1.67 | 3.91 | 3.60 |
| HS8 | 0 | 2.77 | 3.81 | 4.06 |
| HS9 | 0 | 2.34 | 4.06 | 3.91 |
| HS10 | 0 | 2.49 | 3.82 | 3.74 |
| HS11 | 0 | 1.85 | 3.76 | 4.46 |
| HS12 | 0 | 0.53 | 2.09 | 1.74 |
| HS13 | 0 | 2.53 | 4.54 | 3.97 |
| HS14 | 0 | 3.22 | 4.84 | 4.91 |
| HS15 | 0 | 2.40 | 3.83 | 4.06 |
| HS16 | 0 | 2.73 | 4.10 | 4.28 |
| HS17 | 0 | 1.63 | 3.49 | 3.54 |
| HS18 | 0 | 2.07 | 3.54 | 4.15 |
| HS19 | 0 | 2.78 | 4.17 | 4.42 |
| HS20 | 0 | 5.42 | 2.92 | 8.12 |
| HS21 | 0 | 3.43 | 4.54 | 5.08 |
| HS22 | 0 | 0.77 | 2.56 | 2.64 |
| HS23 | 0 | 2.93 | 4.60 | 5.36 |
| HS24 | 0 | 2.78 | 4.61 | 4.71 |
| HS25 | 0 | 2.11 | 3.24 | 3.80 |
| HS26 | 0 | 3.28 | 3.25 | 5.24 |
| HS27 | 0 | 1.39 | 3.09 | 3.24 |
| HS28 | 0 | 2.05 | 3.04 | 3.59 |
| HS29 | 0 | 2.12 | 3.94 | 3.97 |
| HS30 | 0 | 1.93 | 3.07 | 3.81 |
| HS31 | 0 | 3.26 | 4.47 | 4.92 |
| HS32 | 0 | 2.50 | 3.93 | 4.76 |
| HS33 | 0 | 2.79 | 4.47 | 4.48 |
| HS34 | 0 | 1.59 | 3.10 | 3.48 |
| HS35 | 0 | 3.24 | 4.23 | 4.83 |
| HS36 | 0 | 2.88 | 4.25 | 4.59 |
| HS37 | 0 | 2.13 | 3.62 | 3.71 |
| HS38 | 0 | 1.89 | 3.35 | 3.58 |
| HS39 | 0 | 2.35 | 3.69 | 4.04 |
| HS40 | 0 | 2.31 | 3.83 | 3.93 |
| HS41 | 0 | 2.68 | 4.03 | 4.73 |
| HS42 | 0 | 1.44 | 3.24 | 3.61 |
| HS43 | 0 | 2.30 | 3.86 | 4.09 |
| HS44 | 0 | 2.77 | 4.79 | 4.16 |
| HS45 | 0 | 0.21 | 2.17 | 1.48 |
|  |  |  |  |  |

**Supplemental Table S6 (continued a):** **Normalized Cq values (ΔCqs) of the candidate miRNAs investigated for the association with T1DMin the Manchester cohort.**

| Patient ID | incident T1DM  (0=no, 1=yes) | miR-192 (ΔCq) | miR-194 (ΔCq) | miR-215 (ΔCq) |
| --- | --- | --- | --- | --- |
| HS48 | 0 | 2.15 | 3.68 | 3.73 |
| HS49 | 0 | 1.25 | 3.19 | 3.03 |
| HS48 | 0 | 2.15 | 3.68 | 3.73 |
| HS49 | 0 | 1.25 | 3.19 | 3.03 |
| HS50 | 0 | 1.76 | 3.06 | 4.06 |
| HS51 | 0 | 1.20 | 2.64 | 2.85 |
| HS52 | 0 | 2.65 | 4.00 | 4.89 |
| HS53 | 0 | 2.84 | 4.02 | 5.06 |
| HS54 | 0 | 1.75 | 3.39 | 3.60 |
| DM1-1 | 1 | 4.87 | 4.86 | 5.72 |
| DM1-2 | 1 | 1.50 | 3.27 | 2.61 |
| DM1-3 | 1 | 2.12 | 4.84 | 4.25 |
| DM1-7 | 1 | 2.02 | 3.50 | 4.04 |
| DM1-8 | 1 | 2.37 | 3.66 | 3.86 |
| DM1-10 | 1 | 1.17 | 2.99 | 2.79 |
| DM1-11 | 1 | 2.13 | 4.46 | 5.56 |
| DM1-12 | 1 | 2.18 | 4.34 | 4.50 |
| DM1-13 | 1 | 1.05 | 2.58 | 2.76 |
| DM1-14 | 1 | 1.39 | 3.72 | 2.63 |
| DM1-15 | 1 | 1.50 | 3.49 | 3.50 |
| DM1-16 | 1 | 2.89 | 3.89 | 4.41 |
| DM1-17 | 1 | 1.16 | 2.69 | 3.11 |
| DM1-18 | 1 | 2.50 | 3.86 | 4.42 |
| DM1-19 | 1 | 1.36 | 3.06 | 3.31 |
| DM1-20 | 1 | 1.09 | 2.48 | 2.98 |
| DM1-21 | 1 | 1.00 | 2.92 | 4.07 |
| DM1-22 | 1 | -1.17 | 0.28 | 1.03 |
| DM1-23 | 1 | 2.99 | 3.64 | 4.52 |
| DM1-24 | 1 | 1.56 | 3.57 | 3.48 |
| DM1-25 | 1 | 2.15 | 2.47 | 3.25 |
| DM1-26 | 1 | 1.24 | 2.89 | 3.70 |
| DM1-27 | 1 | 2.44 | 3.53 | 3.87 |
| DM1-28 | 1 | 2.44 | 4.14 | 4.04 |
| DM1-29 | 1 | 1.49 | 3.19 | 3.23 |
| DM1-30 | 1 | 2.59 | 3.34 | 4.20 |
| DM1-31 | 1 | 2.12 | 2.55 | 4.25 |
| DM1-32 | 1 | 1.88 | 3.54 | 3.50 |
| DM1-33 | 1 | 1.51 | 2.68 | 3.41 |
| DM1-34 | 1 | 1.94 | 3.13 | 4.10 |
| DM1-35 | 1 | 1.86 | 3.77 | 3.68 |
| DM1-36 | 1 | 2.82 | 3.25 | 4.58 |
|  |  |  |  |  |

**Supplemental Table S6 (continued b):** **Normalized Cq values (ΔCqs) of the candidate miRNAs investigated for the association with T1DM in the Manchester cohort.**

| Patient ID | incident T1DM  (0=no, 1=yes) | miR-192 (ΔCq) | miR-194 (ΔCq) | miR-215 (ΔCq) |
| --- | --- | --- | --- | --- |
| DM1-37 | 1 | 1.80 | 2.74 | 3.34 |
| DM1-38 | 1 | 1.07 | 2.73 | 2.80 |
| DM1-39 | 1 | 2.78 | 4.01 | 4.49 |
| DM1-40 | 1 | 2.67 | 3.67 | 4.98 |
| DM1-S1 | 1 | 1.53 | 2.76 | 2.93 |
| DM1-S2 | 1 | -1.01 | 1.08 | 0.62 |
| DM1-S3 | 1 | 1.10 | 2.33 | 3.71 |
| DM1-S4 | 1 | 1.13 | 3.01 | 2.90 |
| DM1-S5 | 1 | 2.11 | 3.74 | 3.63 |
| DM1-S6 | 1 | 1.92 | 3.43 | 3.46 |
| DM1-S7 | 1 | 2.02 | 3.90 | 3.78 |
| DM1-S8 | 1 | 2.70 | 4.49 | 3.74 |
| DM1-S9 | 1 | 1.76 | 3.03 | 3.61 |
| DM1-S10 | 1 | 2.49 | 4.17 | 3.76 |
| DM1-S11 | 1 | 2.26 | 3.69 | 3.68 |
| DM1-S12 | 1 | 0.83 | 2.18 | 2.77 |
| DM1-S13 | 1 | 2.10 | 3.96 | 3.86 |
| DM1-S14 | 1 | 1.45 | 3.29 | 3.25 |
| DM1-S15 | 1 | 2.00 | 2.80 | 3.91 |
| DM1-S16 | 1 | 1.73 | 3.45 | 4.15 |
| DM1-S17 | 1 | 1.95 | 3.64 | 4.32 |
| DM1-S18 | 1 | 2.96 | 4.80 | 4.62 |
|  |  |  |  |  |

By means of individual qRT-PCR assays (Exiqon) we analyzed sera of 53 patients with normal fasting glucose and 54 patients with type 1 diabetes. Circulating miRs were recorded at baseline. The candidate miRs were normalized to the mean expression of miR-103, miR-106a and miR-425. T1DM: manifest type 1 diabetes mellitus.

**Supplemental Table S7: Characteristics of the longitudinal validation cohort (VIVIT study)**

|  | **no incident T2DM (n=178)** | | **incident T2DM (n=35)** | | ***P*-value** |
| --- | --- | --- | --- | --- | --- |
| Sex (female) | 56 | (33%) | 7 | (20%) | 0.162 |
| Age [years] | 62 | 56-69 | 61 | 52-67 | 0.654 |
| MetS (ATPIII) | 35 | (20%) | 12 | (34%) | 0.080 |
| BMI | 26.2 | 24.3-29.5 | 27.7 | 25.2-30.9 | 0.063 |
| **Fasting glucose [mmol/l]** | **5.2** | **4.9-5.6** | **5.7** | **5.3-6.2** | **<0.001** |
| **HbA1c [%] *** | **5.6** | **5.4-5.9** | **5.9** | **5.6-6.1** | **0.001** |
| **C-Peptide [µg/l]** | **2.0** | **1.4-2.6** | **2.7** | **2.3-3.1** | **<0.001** |
| Insulin [mU/l] | 7.7 | 4.8-10.8 | 8.1 | 5.8-13.9 | 0.403 |
| Creatinine [µmol/l] | 88 | 80-97 | 88 | 71-97 | 0.961 |
| Cystatine C [mg/l] | 0.9 | 0.8-1.1 | 0.9 | 0.8-1.1 | 0.187 |
| eGFR [ml/min/1.73 m2] |  |  |  |  |  |
| CKD-EPI (Creatinine) | 76 | 67-89 | 79 | 69-88 | 0.430 |
| CKD-EPI (Cystatine C) | 80 | 67-95 | 88 | 67-101 | 0.185 |
| CKD-EPI (Crea + Cys C) | 87 | 75-103 | 92 | 69-107 | 0.651 |
| **Triacylclycerol [mmol‎/l]** | **1.4** | **1.0-1.9** | **2.0** | **1.2-2.7** | **0.037** |
| Cholesterol [mmol‎/l] | 5.4 | 4.6-5.9 | 5.5 | 4.8-6.6 | 0.383 |
| HDL [mmol‎/l] | 1.3 | 1.1-1.6 | 1.2 | 1.0-1.5 | 0.116 |
| LDL [mmol‎/l] | 3.5 | 2.9-4.1 | 3.5 | 2.5-4.6 | 0.838 |
| AST [U/l] | 24 | 21-29 | 23 | 20-27 | 0.386 |
| ALT [U/l] | 9 | 41426 | 10 | 42125 | 0.767 |
| GGT [U/l] | 29 | 21-51 | 43 | 23-56 | 0.275 |
| Significant coronary stenoses ≥50% | 100 | (56%) | 24 | (69%) | 0.120 |
| Myocardial infarction | 50 | (28%) | 12 | (34%) | 0.292 |
| Stroke | 4 | (2%) | 3 | (9%) | 0.089 |

Continuous variables are presented as medians with interquartile ranges; Categorical variables are presented as numbers and percentages. *P*-values were calculated using the Mann-Withney U test for continuous variables and the χ^2^ test for categorical variables. Parameters with significant differences are shown in bold font. * To convert % HbA1c to mmol/mol, multiply by 10.9 and subtract 23.5.

**Supplemental Table S8:** **Normalized Cq values (ΔCqs) of the candidate miRNAs investigated for their power to predict future T2DM (VIVIT study).**

| Patient ID | incident T2DM (0=no, 1=yes) | miR-122 (ΔCq) | miR-192 (ΔCq) | miR-194 (ΔCq) | miR-215 (ΔCq) |
| --- | --- | --- | --- | --- | --- |
| 5 | 1 | 0.46 | 1.87 | 2.88 | 5.09 |
| 14 | 1 | -1.25 | 0.98 | 1.30 | 4.28 |
| 21 | 0 | 2.36 | 2.82 | 3.26 | 4.50 |
| 22 | 0 | 2.77 | 2.78 | 3.11 | 3.71 |
| 24 | 0 | 1.02 | 2.35 | 3.10 | 4.16 |
| 25 | 0 | 2.11 | 1.90 | 3.16 | 4.86 |
| 27 | 0 | 0.35 | 2.81 | 2.96 | 4.99 |
| 30 | 1 | 3.62 | 2.64 | 3.98 | 3.66 |
| 32 | 1 | 0.84 | 1.42 | 3.30 | 3.80 |
| 35 | 0 | 0.63 | 2.09 | 2.56 | 3.39 |
| 38 | 0 | -0.41 | 1.33 | 2.16 | 2.45 |
| 45 | 1 | -0.15 | 1.58 | 0.00 | 4.82 |
| 47 | 0 | 0.60 | 2.70 | 2.97 | 3.68 |
| 48 | 1 | -0.42 | 2.07 | 2.52 | 3.38 |
| 53 | 0 | 0.65 | 2.97 | 1.86 | 4.10 |
| 56 | 0 | 1.83 | 2.28 | 3.97 | 3.50 |
| 57 | 0 | 1.57 | 1.95 | 2.89 | 3.48 |
| 70 | 0 | 1.09 | 1.42 | 3.42 | 3.60 |
| 71 | 0 | -0.24 | 1.69 | 2.81 | 2.99 |
| 74 | 1 | 3.32 | 2.41 | 4.30 | 4.94 |
| 76 | 0 | 0.65 | 0.99 | 2.65 | 2.53 |
| 77 | 0 | 4.73 | 5.49 | 4.48 | 4.91 |
| 79 | 0 | 0.00 | 1.16 | 2.20 | 2.35 |
| 80 | 0 | 2.07 | 3.11 | 3.93 | 4.53 |
| 85 | 0 | -1.19 | 0.84 | 1.85 | 2.62 |
| 91 | 0 | 3.49 | 3.13 | 3.39 | 3.72 |
| 100 | 0 | 1.13 | 2.96 | 3.74 | 4.43 |
| 105 | 0 | 1.93 | 2.90 | 2.80 | 4.27 |
| 108 | 0 | -1.97 | 0.75 | 1.84 | 2.11 |
| 109 | 0 | -2.13 | 0.41 | 1.37 | 2.25 |
| 112 | 0 | 1.66 | 2.50 | 3.20 | 4.45 |
| 117 | 0 | 1.00 | 2.43 | 3.55 | 4.39 |
| 119 | 0 | 1.26 | 1.03 | 3.65 | 4.62 |
| 120 | 0 | 2.08 | 1.91 | 3.51 | 4.56 |
| 122 | 0 | 0.18 | 3.61 | 2.98 | 4.96 |
| 123 | 0 | -0.01 | 2.63 | 2.66 | 4.31 |
| 132 | 0 | 0.24 | 2.23 | 3.99 | 2.53 |
| 136 | 0 | 1.47 | 2.10 | 3.04 | 3.72 |

**Supplemental Table S7 (continued a):** **Normalized Cq values (ΔCqs) of the candidate miRNAs investigated for their power to predict future T2DM (VIVIT study).**

| Patient ID | incident T2DM (0=no, 1=yes) | miR-122 (ΔCq) | miR-192 (ΔCq) | miR-194 (ΔCq) | miR-215 (ΔCq) |
| --- | --- | --- | --- | --- | --- |
| 138 | 1 | -0.77 | 0.62 | 2.48 | 3.26 |
| 145 | 0 | -0.16 | 2.08 | 2.58 | 3.07 |
| 147 | 1 | 1.60 | 2.21 | 3.76 | 3.77 |
| 148 | 1 | 1.59 | 1.79 | 2.57 | 4.69 |
| 155 | 0 | -0.29 | 1.99 | 2.58 | 3.30 |
| 157 | 0 | 2.11 | 2.73 | 2.65 | 5.12 |
| 164 | 0 | 1.43 | 2.79 | 5.37 | 4.34 |
| 166 | 0 | 0.96 | 2.23 | 3.46 | 4.02 |
| 179 | 0 | 0.63 | 1.45 | 4.46 | 3.71 |
| 180 | 0 | 2.36 | 2.44 | 3.91 | 4.83 |
| 181 | 0 | 2.91 | 3.96 | 5.62 | 4.74 |
| 184 | 1 | -1.21 | -0.44 | 1.26 | 2.80 |
| 185 | 0 | 0.97 | 2.91 | 3.04 | 4.88 |
| 194 | 0 | 4.41 | 3.49 | 6.56 | 5.19 |
| 196 | 0 | 0.36 | 2.87 | 2.71 | 4.08 |
| 200 | 0 | 2.01 | 2.47 | 4.10 | 4.04 |
| 201 | 0 | -0.08 | 1.22 | 2.64 | 2.65 |
| 205 | 1 | 0.74 | 2.40 | 3.09 | 4.11 |
| 207 | 0 | 2.57 | 2.21 | 3.32 | 4.26 |
| 208 | 0 | 1.24 | 2.71 | 3.96 | 4.25 |
| 215 | 0 | 0.07 | 3.51 | 3.44 | 4.44 |
| 216 | 0 | 0.61 | 2.55 | 3.04 | 3.10 |
| 218 | 0 | 0.71 | 1.43 | 3.06 | 3.00 |
| 219 | 0 | 1.99 | 1.83 | 2.92 | 3.73 |
| 227 | 0 | -0.50 | 1.90 | 2.69 | 4.56 |
| 229 | 0 | 0.60 | 2.96 | 3.43 | 3.67 |
| 230 | 0 | 2.84 | 3.06 | 3.58 | 4.15 |
| 232 | 0 | -0.11 | 1.92 | 2.51 | 3.12 |
| 233 | 0 | 2.10 | 3.33 | 3.70 | 4.39 |
| 235 | 0 | -1.50 | 1.69 | 1.28 | 3.20 |
| 237 | 0 | 0.00 | 1.65 | 2.85 | 3.86 |
| 238 | 1 | -0.24 | 2.36 | 2.62 | 3.34 |
| 256 | 0 | 1.24 | 1.68 | 4.74 | 5.49 |
| 259 | 0 | 1.14 | 3.11 | 3.35 | 4.46 |
| 262 | 0 | 1.41 | 3.01 | 3.91 | 4.75 |
| 267 | 0 | 0.43 | 2.54 | 3.06 | 4.64 |
| 270 | 1 | 1.25 | 1.81 | 2.70 | 4.44 |
| 272 | 0 | -0.15 | 1.51 | 2.55 | 2.78 |
| 282 | 1 | 2.41 | 1.79 | 3.52 | 4.43 |
| 291 | 0 | 1.12 | 1.62 | 2.88 | 4.44 |
| 296 | 0 | -3.63 | -0.05 | 1.16 | 1.33 |
| 301 | 0 | 2.42 | 3.00 | 3.60 | 5.20 |
| 304 | 1 | 0.55 | 1.12 | 2.51 | 3.21 |
| 305 | 0 | -1.98 | 1.40 | 1.88 | 3.08 |
| 309 | 1 | 0.56 | 1.62 | 2.82 | 3.58 |
| 319 | 1 | 3.48 | 3.39 | 4.09 | 3.76 |
| 320 | 0 | 0.68 | 2.38 | 3.26 | 0.98 |
| 324 | 1 | 1.66 | 2.42 | 2.89 | 3.56 |

| **Supplemental Table S7 (continued b):** **Normalized Cq values (ΔCqs) of the candidate miRNAs investigated for their power to predict future T2DM (VIVIT study).** | | | | | |
| --- | --- | --- | --- | --- | --- |
| Patient ID | incident T2DM (0=no, 1=yes) | miR-122 (ΔCq) | miR-192 (ΔCq) | miR-194 (ΔCq) | miR-215 (ΔCq) |
| 330 | 0 | 0.24 | 2.37 | 3.28 | 4.39 |
| 331 | 0 | -0.42 | -0.33 | 0.60 | 3.96 |
| 333 | 0 | 1.31 | 3.04 | 3.43 | 4.17 |
| 334 | 1 | 2.33 | 3.22 | 4.08 | 5.89 |
| 338 | 0 | -0.52 | 1.70 | 2.76 | 4.01 |
| 339 | 0 | 1.85 | 3.99 | 3.76 | 4.31 |
| 340 | 0 | 0.23 | 1.92 | 2.57 | 4.70 |
| 342 | 0 | 0.90 | 2.41 | 3.26 | 3.50 |
| 348 | 0 | 0.72 | 2.99 | 3.66 | 3.70 |
| 353 | 0 | 0.05 | 2.21 | 3.07 | 3.62 |
| 354 | 0 | 0.90 | 2.41 | 3.26 | 3.50 |
| 355 | 0 | 0.72 | 2.99 | 3.66 | 3.70 |
| 362 | 1 | 1.24 | 1.71 | 2.55 | 4.49 |
| 371 | 0 | 0.24 | 2.65 | 3.36 | 4.15 |
| 372 | 0 | 1.20 | 3.11 | 4.12 | 5.27 |
| 376 | 0 | 0.77 | 2.99 | 3.32 | 3.72 |
| 380 | 0 | 0.85 | 0.88 | 1.99 | 5.23 |
| 382 | 0 | 2.20 | 3.20 | 4.17 | 4.89 |
| 385 | 0 | 0.64 | 2.93 | 3.80 | 4.11 |
| 391 | 0 | 2.71 | 3.57 | 4.68 | 4.76 |
| 396 | 0 | 1.30 | 1.90 | 3.17 | 5.16 |
| 397 | 0 | 2.07 | 2.44 | 3.15 | 4.26 |
| 406 | 0 | 1.00 | 2.05 | 3.61 | 3.35 |
| 408 | 0 | 1.35 | 0.80 | 1.94 | 4.87 |
| 411 | 0 | 0.74 | 1.91 | 2.75 | 3.54 |
| 412 | 0 | 2.01 | 2.45 | 3.34 | 3.42 |
| 413 | 0 | 3.14 | 3.13 | 3.57 | 3.83 |
| 414 | 0 | 1.35 | 0.80 | 1.94 | 4.87 |
| 420 | 0 | -1.58 | 1.25 | 2.71 | 2.87 |
| 421 | 1 | 1.85 | 2.10 | 3.30 | 4.03 |
| 429 | 1 | 2.68 | 2.31 | 3.30 | 4.42 |
| 431 | 0 | 0.79 | 2.55 | 3.95 | 3.76 |
| 432 | 0 | 0.30 | 0.53 | 3.25 | 3.92 |
| 433 | 0 | 1.90 | 3.51 | 3.32 | 4.07 |
| 436 | 0 | 1.46 | 3.17 | 3.97 | 4.44 |
| 437 | 1 | -1.63 | 1.62 | 2.38 | 2.55 |
| 444 | 1 | 0.81 | 2.65 | 2.88 | 4.38 |
| 448 | 0 | 0.05 | 1.00 | 2.30 | 2.75 |
| 449 | 0 | 0.35 | 1.63 | 2.99 | 3.00 |
| 460 | 0 | 2.65 | 2.52 | 3.09 | 4.61 |
| 462 | 0 | 3.18 | 3.25 | 4.47 | 5.16 |
| 463 | 0 | 0.63 | 1.79 | 3.15 | 3.23 |
| 464 | 0 | 1.55 | 2.66 | 2.49 | 5.07 |
| 472 | 1 | -1.73 | -1.38 | 0.87 | 3.18 |
| 492 | 0 | 0.87 | 2.18 | 3.66 | 4.33 |
| 494 | 0 | -0.19 | 2.44 | 3.05 | 4.20 |
| 495 | 0 | 0.64 | 2.21 | 2.98 | 3.41 |

| **Supplemental Table S7 (continued c):** **Normalized Cq values (ΔCqs) of the candidate miRNAs investigated for their power to predict future T2DM (VIVIT study).** |
| --- |

| Patient ID | incident T2DM (0=no, 1=yes) | miR-122 (ΔCq) | miR-192 (ΔCq) | miR-194 (ΔCq) | miR-215 (ΔCq) |
| --- | --- | --- | --- | --- | --- |
| 497 | 0 | 2.41 | 3.74 | 4.85 | 4.78 |
| 498 | 0 | 3.64 | 4.56 | 5.13 | 4.46 |
| 499 | 0 | -0.46 | 2.20 | 3.50 | 3.78 |
| 500 | 0 | 0.70 | 2.89 | 3.04 | 4.48 |
| 501 | 0 | -0.35 | 1.45 | 2.16 | 3.13 |
| 508 | 1 | 1.22 | 2.01 | 2.95 | 3.55 |
| 515 | 0 | 1.31 | 2.70 | 3.50 | 4.60 |
| 516 | 0 | 0.37 | 2.60 | 3.10 | 5.61 |
| 520 | 0 | 2.31 | 2.38 | 3.08 | 4.07 |
| 527 | 0 | 0.87 | 2.16 | 3.45 | 3.54 |
| 530 | 0 | 2.07 | 2.04 | 3.15 | 4.54 |
| 537 | 1 | -3.01 | -0.86 | 0.53 | 4.63 |
| 543 | 0 | 1.37 | 2.40 | 3.28 | 4.20 |
| 553 | 0 | 1.62 | 2.10 | 2.45 | 5.54 |
| 557 | 1 | -0.05 | 1.22 | 2.37 | 4.53 |
| 558 | 0 | -0.81 | 1.52 | 2.14 | -1.64 |
| 559 | 0 | 0.43 | 2.45 | 2.12 | 3.10 |
| 564 | 0 | 0.27 | 1.40 | 2.29 | 2.99 |
| 574 | 0 | 2.92 | 3.03 | 3.84 | 4.02 |
| 580 | 0 | 1.93 | 2.90 | 2.80 | 4.27 |
| 582 | 1 | -0.27 | 2.07 | 3.40 | 3.41 |
| 587 | 0 | -0.62 | 1.67 | 2.10 | 4.82 |
| 588 | 0 | 1.22 | 5.49 | 4.66 | 3.95 |
| 589 | 0 | 1.41 | 3.27 | 3.22 | 4.50 |
| 590 | 0 | 2.38 | 3.70 | 4.47 | 1.97 |
| 592 | 0 | -0.64 | 1.38 | 2.11 | 4.26 |
| 593 | 0 | 1.73 | 3.36 | 3.43 | 4.79 |
| 596 | 0 | 0.38 | 2.78 | 3.41 | 4.61 |
| 599 | 0 | 1.44 | 2.04 | 3.99 | 3.90 |
| 601 | 0 | 0.29 | 2.34 | 2.89 | 4.90 |
| 607 | 0 | 0.80 | 2.92 | 4.01 | 4.31 |
| 610 | 0 | 1.87 | 3.92 | 5.25 | 5.01 |
| 611 | 0 | 0.00 | 2.01 | 2.57 | 4.39 |
| 617 | 0 | 0.05 | 1.84 | 2.58 | 5.38 |
| 626 | 0 | 0.62 | 3.39 | 3.00 | 5.25 |
| 629 | 0 | 1.25 | 2.94 | 4.66 | 3.89 |
| 630 | 0 | -0.79 | 2.28 | 4.33 | 3.92 |
| 631 | 0 | 1.09 | 2.89 | 4.11 | 4.31 |
| 632 | 0 | -0.14 | 0.75 | 2.35 | 3.49 |
| 635 | 0 | 1.64 | 2.75 | 5.34 | 4.26 |
| 641 | 0 | 1.32 | 3.23 | 4.60 | 4.99 |
| 652 | 1 | 1.58 | 2.57 | 1.77 | 5.09 |
| 668 | 0 | 1.65 | 2.56 | 3.27 | 4.78 |
| 673 | 0 | 0.71 | 1.58 | 2.72 | 3.89 |
| 680 | 0 | -1.04 | 1.68 | 2.06 | 4.10 |
| 691 | 0 | 1.56 | 2.37 | 2.38 | 4.67 |
| 694 | 0 | 0.91 | 2.40 | 3.76 | 4.84 |

| **Supplemental Table S7 (continued d):** **Normalized Cq values (ΔCqs) of the candidate miRNAs investigated for their power to predict future T2DM (VIVIT study).** | | | | | |
| --- | --- | --- | --- | --- | --- |
|  | | | | | |
| Patient ID | incident T2DM (0=no, 1=yes) | miR-122 (ΔCq) | miR-192 (ΔCq) | miR-194 (ΔCq) | miR-215 (ΔCq) |
| 696 | 0 | 2.63 | 3.59 | 5.81 | 4.01 |
| 697 | 0 | -1.26 | 2.48 | 2.36 | 4.22 |
| 698 | 0 | -0.10 | 1.87 | 2.59 | 4.20 |
| 701 | 1 | 2.59 | 2.58 | 3.37 | 4.24 |
| 702 | 0 | 1.88 | 2.63 | 2.77 | 4.95 |
| 710 | 0 | 1.57 | 2.75 | 2.74 | 5.43 |
| 715 | 0 | -0.92 | 3.85 | 4.81 | 3.98 |
| 722 | 0 | 1.56 | 2.38 | 2.67 | 3.72 |
| 728 | 0 | 0.18 | 3.50 | 4.29 | 5.16 |
| 731 | 0 | 0.32 | 2.01 | 2.64 | 4.07 |
| 740 | 1 | 2.82 | 2.53 | 2.93 | 5.22 |
| 741 | 0 | 1.77 | 1.71 | 2.43 | 4.17 |
| 742 | 0 | 1.92 | 2.85 | 4.00 | 6.12 |
| 749 | 1 | 1.22 | 2.30 | 2.47 | 4.48 |
| 757 | 0 | 0.46 | 2.97 | 4.30 | 4.87 |
| 759 | 0 | 1.29 | 3.83 | 3.99 | 5.81 |
| 760 | 0 | 1.87 | 2.60 | 4.16 | 4.81 |
| 763 | 0 | 0.57 | 2.79 | 3.41 | 5.78 |
| 764 | 0 | -1.96 | 1.19 | 1.39 | 2.78 |
| 768 | 0 | -0.35 | 2.80 | 3.15 | 4.12 |
| 770 | 0 | 0.85 | 2.73 | 2.73 | 3.74 |
| 773 | 0 | -0.49 | 2.61 | 3.34 | 3.89 |
| 776 | 0 | 1.02 | 3.31 | 4.34 | 4.64 |
| 778 | 0 | 3.25 | 3.44 | 4.42 | 4.75 |
| 782 | 0 | -2.38 | 0.16 | 1.13 | 1.99 |
| 783 | 0 | 1.63 | 3.96 | 4.46 | 4.91 |
| 786 | 0 | 2.71 | 2.98 | 3.58 | 4.02 |
| 788 | 0 | 0.70 | 3.13 | 3.34 | 4.17 |
| 793 | 1 | -1.09 | -0.02 | 1.04 | 4.42 |
| 794 | 0 | 0.54 | 1.78 | 2.60 | 3.65 |
| 795 | 0 | 0.85 | 2.51 | 4.09 | 3.92 |
| 796 | 0 | -0.09 | 1.18 | 2.69 | 3.59 |
| 803 | 0 | -3.02 | 0.17 | 1.22 | 1.02 |
|  |  |  |  |  |  |
| Median  (IQR) | 0 | 0.87  (0.18-1.76) | 2.45  (1.88-2.97) | 3.19  (2.66-3.83) | 4.15  (3.59-4.69) |
| Median  (IQR) | 1 | 0.84  (-0.27-1.85) | 2.01  (1.43-2.419 | 2.82  (2.38-3.30) | 4.24  (3.55-4.53) |
| P-value |  | 0.822 | <0.001 | 0.003 | 0.996 |
|  |  |  |  |  |  |

By means of individual qRT-PCR assays (Exiqon) we analyzed sera of 35 patients who developed diabetes during the 6-year follow-up (incident T2DM) and 178 individuals who did not (no incident T2DM). Circulating miRs were recorded at baseline. The candidate miRs were normalized to the mean expression of miR-103, miR-106a and miR-425. P-values were calculated using the Mann-Withney U test. Incident T2DM, incident type 2 diabetes mellitus.

**Supplemental Table S9: Multivariate logistic regression models with classical predictors (Model A and B) and with cardiovascular disease (Model C and D) (VIVIT study).**

|  | Standardized covariates | OR | (95% CI) | *P*-value | *Nagelkerke R^2^ (%)* |
| --- | --- | --- | --- | --- | --- |
| Model A | Sex (female) | 0.53 | (0.21-1.34) | 0.182 | 13.0 |
|  | Age | 1.18 | (0.78-1.79) | 0.429 |  |
|  | MetS (ATPIII) | 1.89 | (0.82-4.38) | 0.138 |  |
|  | **miR-192** | **1.93** | **(1.30-2.85)** | **0.001** |  |
| Model B | Sex (female) | 0.54 | (0.22-1.36) | 0.191 | 12.2 |
|  | Age | 1.18 | (0.78-1.78) | 0.442 |  |
|  | MetS (ATPIII) | 2.15 | (0.93-4.98) | 0.075 |  |
|  | **miR-194** | **1.90** | **(1.27-2.84)** | **0.002** |  |
| Model C | Significant coronary stenosis ≥50% | 2.155 | (0.88-5.31) | 0.095 | 14.6 |
|  | Myocardial infarction | 0.895 | (0.37-2.16) | 0.806 |  |
|  | Stroke | 4.209 | (0.85-20.83) | 0.078 |  |
|  | **miR-192** | **2.111** | **(1.42-3.14)** | **<0.001** |  |
| Model D | Significant coronary stenosis ≥50% | 2.332 | (0.95-5.75) | 0.066 |  |
|  | Myocardial infarction | 0.752 | (0.31-1.85) | 0.534 |  |
|  | Stroke | 3.573 | (0.73-17.44) | 0.115 |  |
|  | **miR-194** | **2.060** | **(1.35-3.18)** | **0.001** |  |

Multivariate logistic regression results show that miR-192 (Model A and C) and miR-194 (Model B and D) remain significant predictors for the development of diabetes after adjustment for classical predictors and cardiovascular disease. Variables are log transformed if necessary. Continuous variables were standardized in SD units. ORs of the miRs are calculated with - ΔCt values, because ΔCt values have an inverse correlation to increasing miR concentrations. MetS (ATPIII), metabolic syndrome (adult treatment pannel III definition); SD, standard deviation; OR, odds ratio.

**References of Supplementary Information**

1. Vandesompele J, De Preter K, Pattyn F, et al. (2002) Accurate normalization of real-time quantitative RT-PCR data by geometric averaging of multiple internal control genes. Genome Biol 3: RESEARCH0034
2. Andersen CL, Jensen JL, Orntoft TF (2004) Normalization of real-time quantitative reverse transcription-PCR data: a model-based variance estimation approach to identify genes suited for normalization, applied to bladder and colon cancer data sets. Cancer Res 64: 5245-5250
3. Ortega FJ, Mercader JM, Moreno-Navarrete JM, Rovira O, Guerra E, Esteve E, Xifra G, Martinez C, Ricart W, Rieusset J, Rome S, Karczewska-Kupczewska M, Straczkowski M, Fernandez-Real JM: Profiling of circulating microRNAs reveals common microRNAs linked to type 2 diabetes that change with insulin sensitization. Diabetes Care 2014;37:1375-1383
4. Song J, Bai Z, Han W, Zhang J, Meng H, Bi J, Ma X, Han S, Zhang Z: Identification of suitable reference genes for qPCR analysis of serum microRNA in gastric cancer patients. Dig Dis Sci 2012;57:897-904
5. Pescador N, Perez-Barba M, Ibarra JM, Corbaton A, Martinez-Larrad MT, Serrano-Rios M: Serum circulating microRNA profiling for identification of potential type 2 diabetes and obesity biomarkers. PLoS One 2013;8:e77251
6. Becker PP, Rau M, Schmitt J, Malsch C, Hammer C, Bantel H, Mullhaupt B, Geier A: Performance of Serum microRNAs -122, -192 and -21 as Biomarkers in Patients with Non-Alcoholic Steatohepatitis. PLoS One 2015;10:e0142661
7. Bye A, Rosjo H, Aspenes ST, Condorelli G, Omland T, Wisloff U: Circulating microRNAs and aerobic fitness--the HUNT-Study. PLoS One 2013;8:e57496
8. Wang X, Sundquist J, Zoller B, Memon AA, Palmer K, Sundquist K, Bennet L: Determination of 14 circulating microRNAs in Swedes and Iraqis with and without diabetes mellitus type 2. PLoS One 2014;9:e86792
9. Drexel H, Aczel S, Marte T, et al. (2005) Is atherosclerosis in diabetes and impaired fasting glucose driven by elevated LDL cholesterol or by decreased HDL cholesterol? Diabetes Care 28: 101-107
